# Genetic regulation of gene expression and splicing during a 10-year period of human aging

**DOI:** 10.1186/s13059-019-1840-y

**Published:** 2019-11-04

**Authors:** Brunilda Balliu, Matthew Durrant, Olivia de Goede, Nathan Abell, Xin Li, Boxiang Liu, Michael J. Gloudemans, Naomi L. Cook, Kevin S. Smith, David A. Knowles, Mauro Pala, Francesco Cucca, David Schlessinger, Siddhartha Jaiswal, Chiara Sabatti, Lars Lind, Erik Ingelsson, Stephen B. Montgomery

**Affiliations:** 1 Department of Pathology, Stanford University School of Medicine, Stanford, USA; 2 Department of Genetics, Stanford University School of Medicine, Stanford, USA; 3 Department of Medicine, Division of Cardiovascular Medicine, Stanford University School of Medicine, Stanford, USA; 4 Department of Biomedical Data Science, Stanford University School of Medicine, Stanford, USA; 5 Department of Biology, Stanford University School of Medicine, Stanford, USA; 6 Department of Medical Sciences, Uppsala University, Uppsala, Sweden; 7 Dipartimento di Scienze Biomediche, Universita di Sassari, Sassari, Italy; 8 Laboratory of Genetics, National Institute on Aging, Maryland, USA; 9 Stanford Cardiovascular Institute, Stanford University, Stanford, USA; 10 Stanford Diabetes Research Center, Stanford University, Stanford, USA; 11 New York Genome Center, New York, USA

**Keywords:** Aging, Longevity, Gene expression, Alternative splicing, Gene regulation

## Abstract

**Background:**

Molecular and cellular changes are intrinsic to aging and age-related diseases. Prior cross-sectional studies have investigated the combined effects of age and genetics on gene expression and alternative splicing; however, there has been no long-term, longitudinal characterization of these molecular changes, especially in older age.

**Results:**

We perform RNA sequencing in whole blood from the same individuals at ages 70 and 80 to quantify how gene expression, alternative splicing, and their genetic regulation are altered during this 10-year period of advanced aging at a population and individual level. We observe that individuals are more similar to their own expression profiles later in life than profiles of other individuals their own age. We identify 1291 and 294 genes differentially expressed and alternatively spliced with age, as well as 529 genes with outlying individual trajectories. Further, we observe a strong correlation of genetic effects on expression and splicing between the two ages, with a small subset of tested genes showing a reduction in genetic associations with expression and splicing in older age.

**Conclusions:**

These findings demonstrate that, although the transcriptome and its genetic regulation is mostly stable late in life, a small subset of genes is dynamic and is characterized by a reduction in genetic regulation, most likely due to increasing environmental variance with age.

## Background

While an individual’s genome sequence is mostly stable throughout life, gene expression and genetic regulation of expression fluctuate in response to different environmental exposures [1–3]. The impact of aging on gene expression and genetic regulation has been well-studied in model systems, such as yeast, fruit fly, or worm [4, 5], while much less is known about the transient nature of gene regulation and expression in humans. The majority of studies that have been performed in humans have been cross-sectional [6–15]. The few longitudinal studies have either focused on a specific disease or intervention [16–21], looked over a short time span [22], or focused on cell lines [23]. For example, Bryois et al. [22] studied differences in RNA sequencing-based gene expression levels in whole blood from female twins of the UK Adult Twin Registry longitudinally at two time points separated, on average, by 22 months. At each time point, the individuals varied in age from 45 to 80 years old. Harris et al. [23] studied the impact of aging on array-based gene expression levels in lymphoblastoid cell lines derived from members of the Lothian Birth Cohort 1936 at mean ages 70 and 76 years. The impact of aging on genetic regulation of gene expression was not examined in the Harris et al. [23] study. Even less is known about the effect of age on alternative splicing and its genetics, even though changes in alternative splicing have previously been linked to aging-associated phenotypes [14, 24–27]. As a result, a complete picture of the long-term effect of aging on gene expression and splicing and their genetic regulation in humans is still lacking, especially late in life.

Here, we present the first long-term, longitudinal characterization of changes in gene expression and alternative splicing and their genetic regulation as a function of aging late in life. We performed RNA sequencing in whole blood from 65 healthy participants from the Prospective Investigation of Uppsala Seniors (PIVUS) study [28] at both age 70 and 80, a period of the aging process characterized by high morbidity and mortality. We quantified how total and allele-specific gene expression, alternative splicing, and genetic regulation (expression and splicing quantitative trait loci; eQTLs, sQTLs) are altered over this 10-year period.

We observe that individuals are more similar to their own gene expression profiles than profiles of other individuals their own age; 93% of samples cluster with their own measurements at another age. Despite this, we identified hundreds of genes with differential expression and alternative splicing with age, as well as outlying individual trajectories of aging, i.e., individuals with extreme increase or decrease in expression with age. Moreover, we observed a strong correlation of genetic effects on expression between the two ages (*ρ*_*G*_ =.96; median across genes) and that 7.8% of genes were characterized by genetic dysregulation over the two time points. In contrast, overall allelic imbalance within an individual increases with age by 2.69% (median across individuals). Together, these findings indicate that a small subset of genes is characterized by changes in expression and splicing and a reduction in genetic regulation late in life. The strong correlation of genetic effects and the increase in allelic imbalance with age suggests that increasing environmental variance as opposed to decreased genetic variance underlies the reduction in genetic regulation with age.

## Results

Background noise correction, the process of identifying and correcting for major components of expression variability, unrelated to the variable of interest, e.g., RNA library size or cell-type composition, is crucial in (longitudinal) RNA-Seq experiments in order to improve power and avoid false discoveries [29, 30]. We performed extensive background noise correction for each analysis (see the “Materials and methods” section and Additional file 1). Unless otherwise mentioned, all results are based on analyses corrected for measured and inferred components of gene expression variability.

### Population-level age-specific expression across the transcriptome

**Fig. 1:**
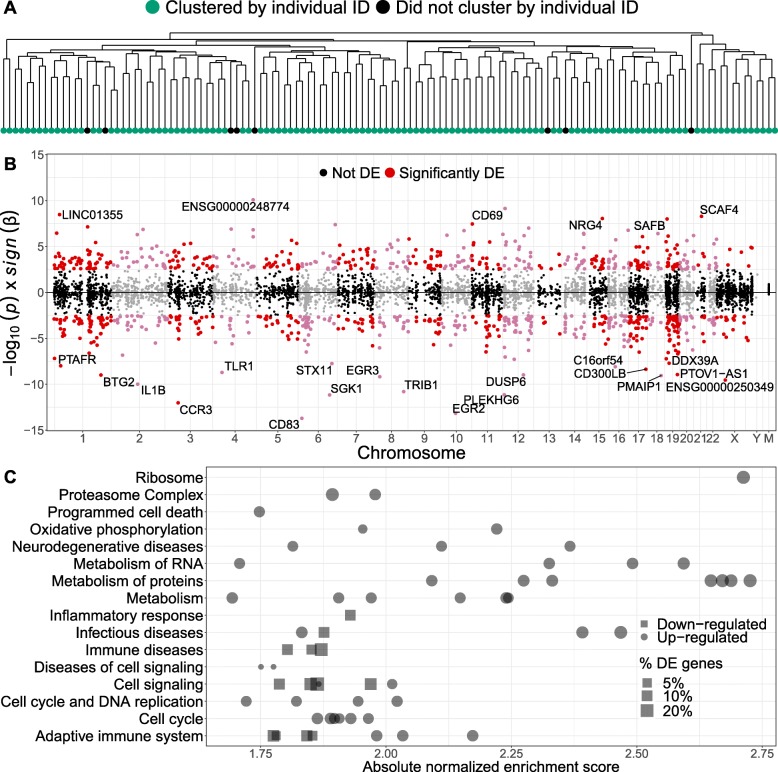
Population-level age-specific expression across the transcriptome. **a** Dendrogram of expression-based sample-to-sample distance. Measurements of the same individual at the two ages cluster together (green) for 93% of samples. **b** Mirror Manhattan plot of the expression-age discoveries. We find 1291 age-associated genes (8% of tested genes; FDR ≤5*%*). Each dot represents a gene; the *x*-axis gives the position of the gene in the genome, and the *y*-axis represents the direction of the age effect and the strength of the association. **c** Gene-set enrichment analysis of genes with age-specific expression. We observe strong enrichment for multiple age-related pathways. Each point represents a pathway; the *x*-axis gives the absolute enrichment score, which reflects the degree to which each pathway is overrepresented at the top or bottom of the ranked list of differentially expressed genes, normalized to account for differences in gene set size and in correlations between gene sets and the expression data set. The *y*-axis lists the parent node of the most enriched pathways (FDR ≤5*%*). The names of each significant pathway are listed in Additional file 3

In order to quantify the stability of gene expression levels within individuals, we measured the correlation of expression across genes between the two time points and performed hierarchical clustering based on the sample-to-sample distance matrix (Fig. 1a and Additional file 1: Figure S4). We identified a moderate correlation of gene expression within an individual across genes (Spearman’s *ρ*=.30; median across individuals; Additional file 1: Figure S4A) and a high similarity of expression profiles; measurements of the same individual at the two ages cluster together for the 93% of samples (Fig. 1a and Additional file 1: Figure S4B).

We investigated transcriptome-wide changes in gene expression with age (Fig. 1b and Additional file 1: Figure S5A and Additional file 2). We discovered 1291 genes (8% of tested genes) whose expression levels were significantly associated with age (FDR ≤ 5*%*). Harris et al. [23] observed a similar number of differentially expressed genes between ages 70 and 76 years in lymphoblastoid cell lines. The DE genes showed significant enrichment (FDR ≤ 5*%*) for multiple aging-related pathways (Fig. 1c and Additional file 3), e.g., metabolism of proteins [31, 32], oxidative phosphorylation [33], and DNA replication [34].

Moreover, 18 of these DE genes are associated with complex traits for which gene expression levels in whole blood modulate disease risk (Additional files 1 and 4). For most of these genes, aging pushes expression toward the direction that is associated with increased disease risk or higher levels of risk factors. For example, the C allele in rs7941030 is associated with reduced expression of *UBASH3B*, which is downregulated with age in our study, and increased cholesterol levels [35]. Thus, aging pushes the expression of *UBASH3B* towards the direction that is associated with increased total cholesterol. For a few genes, aging pushes expression toward the direction of decreased disease risk or risk factor levels, particularly for blood pressure traits. This negative relation between aging and blood pressure could be due to the fact that several of the individuals in our study began using medications that lower blood pressure between age 70 and 80, which in turn results in a significant decrease of both SBP and DBP with age (Wilcoxon signed rank test; *P*_SBP_=0.04; *P*_DBP_=8.25×10^−5^). Other medications, including lipid-lowering, may also induce overlaps between DE genes and complex trait GWAS genes or confound the direction of the effect of aging on expression and complex traits; however, it remains a challenge to disentangle causal relationships based on these results. Finally, it should be noted that most of the traits associated with DE genes are cardio-metabolic, e.g., triglycerides, total cholesterol, and blood pressure. This is likely at least partly due to the larger availability of publicly available well-powered genetic association studies of metabolic traits as compared to other age-related disease (e.g., cancer and neurological diseases).

To further quantify the relative effect of age on gene expression, we estimated the proportion of expression variance explained by age (Additional file 1: Figure S5B). Age explained 1.5% of expression variance for genes significantly associated with age. This estimate is smaller than the estimate from the uncorrected analysis of our data (average across DE genes = 7.9%), due to our conservative background noise correction approach, but comparable to estimates from other aging studies in humans, e.g., 2.2% in Vinuela et al. [14].

We validated our DE genes in silico using summary statistics from two large cross-sectional studies of aging in human PBMCs (CHARGE [11]) and whole blood (SardiNIA[36]). We estimated that 76% and 51% of our DE genes were differentially expressed with age in the CHARGE and SardiNIA cohorts, respectively. In addition, we found a significant overlap between our top 1000 DE genes and the top 1000 DE genes from these other two studies (hyper-geometric exact test for overlap of three sets; *P*=1.3×10^−16^; Additional file 1: Figure S5C). The 49 DE genes that overlap in the top 1000 discoveries between the three studies (Additional file 5) are enriched in gene ontology (GO) terms related to adaptive immune response pathways that have previously been implicated in aging [37], e.g., leukocyte cell-to-cell adhesion and terms reflecting the underlying age-related T cell biology (Additional file 1: Figure S5D). Only 65% of these 49 genes change in the same direction with age across all studies. This discrepancy could be explained by the difference in the age distribution of the samples in the three studies and could reflect non-linear effects of age on expression. It could also be a result of differences in expression assay—CHARGE is array-based, while PIVUS is sequencing-based; or background noise correction method—DE analyses in CHARGE and SardiNIA were only adjusted for measured confounders, while our analyses in PIVUS also adjusted for hidden factors. In addition, 22 and 17 known aging- and longevity-related genes from The Human Aging Genomic Resources [38] GenAge (307 genes) and LongevityMap (212 genes) databases were included in our list of DE genes (Additional file 6).

To study the impact of cell-type composition in our differential expression results, we contrasted our list of DE genes to a list of 547 genes that distinguish 22 human hematopoietic cell phenotypes, including seven T cell types, naïve and memory B cells, plasma cells, NK cells, and myeloid subsets [39]. We found a minimal impact of the cell-type composition in our results; only 4.5% of all DE genes and 5% of top 100 DE genes are signature genes with cell-type-specific expression.

### Individual-level age-specific expression across the transcriptome

**Fig. 2:**
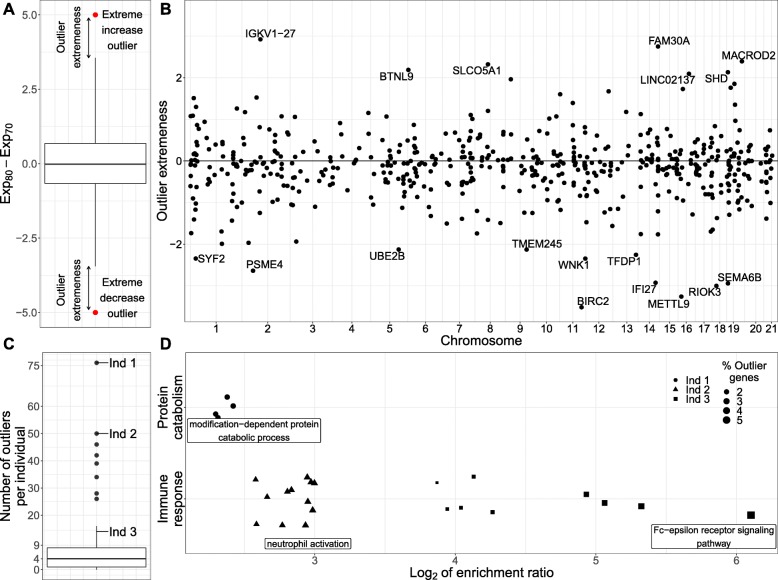
Individual-level age-specific expression across the transcriptome. **a** Illustration of age-trajectory outliers. Individuals are outliers for a gene if their change in the expression of the gene between the two ages falls outside the (*Q*_1_−3×*IQR*, *Q*_3_+3×*IQR*) range, where *Q*_1_ and *Q*_3_ are the 25th and 75th percentiles and IQR is the interquartile range. **b** Mirror Manhattan plot of the age-trajectory outliers. We find 555 individual-gene outlier pairs, consisting of 529 individual genes, 60% of which show extreme expression decrease with age (outlier extremeness < 0). *IGKV1-27*, responsible for antigen binding and involved in adaptive immune response, shows the largest expression increase. *BIRC2*, an apoptosis-inhibitor gene, shows the largest expression decrease. Each dot represents a gene; the *x*-axis gives the position of the gene in the genome, and the *y*-axis represents the outlier extremeness (illustrated in **a**). **c** Number of outlier genes for each individual. The median number of outlier genes per individual was 4 with 14% of individuals showing no outliers while 13% had more than 20 outlier genes. **d** Enrichment for Gene Ontology biological processes for outlier genes of each individual. We observed significant enrichment (FDR ≤5*%*) for specific terms for the outlier genes of three individuals. For two of these individuals, we see enrichment for terms related to immune response. For the third individual, we see enrichment for terms related to protein catabolism

The longitudinal design of our study enabled us to also investigate changes in individual-level expression profiles with age. While such individual-level changes compared to population-level changes can be due to any number of personal environments (i.e., disease, diet, medication), we hypothesized that outlier trajectories may reflect some individual-specific differences in aging. We searched for individuals with outlying age trajectories (schematically illustrated in Fig. 2a) and found 555 individual-gene outlier pairs from 529 unique genes (Fig. 2b and Additional file 7); 60% of which showed outlying decrease in expression with age, as opposed to increase, and 6% of which show also population-level DE with age. Only 5% of the age-trajectory outlier genes are signature genes with cell-type-specific expression, indicating a minimal impact of the cell-type composition in our results. The median number of outlier genes per individual was 4 with 14% of individuals showing no outliers while 13% had more than 20 outlier genes (Fig. 2c). The largest outlying expression increase with age was observed for an individual in *IGKV1-27*, a gene responsible for antigen binding and involved in adaptive immune response. The same individual was an outlier for several additional immunoglobulin-related genes (Additional file 1: Figure S6A). *BIRC2*, a gene which regulates apoptosis and modulates inflammatory signaling and immunity, mitogenic kinase signaling, and cell proliferation, showed the largest expression decrease with age (Additional file 1: Figure S6B). The same individual was an outlier for several other genes related to proteasomal protein catabolic process.

To aid the understanding of the biological basis of personal expression outlier genes, we performed enrichment analysis of GO biological processes for the outlier genes of each individual (Fig. 2d and Additional file 1: Figure S6C) as well as contrast them with outlying covariate/phenotype data for the individual (Additional file 1: Figure S6D). For genes with outlying decrease of expression with age, we observed significant enrichment (FDR ≤ 5*%*) for known age-related GO terms for three individuals. For two of these individuals, one of which showed a large increase in leukocyte counts between the two ages (Additional file 1: Figure S6D), we see enrichment for terms related to immune response [40]. For the third individual, we see enrichment for terms related to protein catabolism [41]. The same individual had a substantial increase in albumin levels between the two ages and was diagnosed with diabetes between age 70 and 80 (Additional file 1: Figure S6D). In contrast, genes in which individuals showed an outlying increase of expression with age were not enriched for any specific functions.

### Stability of genetic regulation of gene expression across the transcriptome with age

**Fig. 3:**
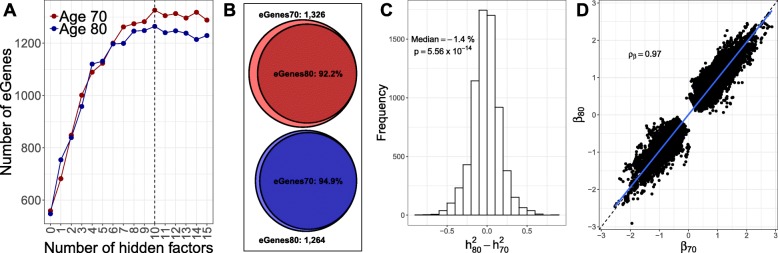
Age-specific genetic regulation across the genome. **a** Number of genes with at least one significant eQTL (eGenes) for uncorrected analysis (0 hidden factors) and analysis corrected for one up to 15 hidden factors. We detected 1326 and 1264 eGenes at age 70 and 80 (FDR ≤ 1*%*), respectively. The depletion of eGenes at age 80, relative to age 70, is statistically significant (exact McNemar’s test; OR = 0.81, *P*=5.8×10^−3^). Dashed line indicates the number of hidden factors that maximizes discovery at each age. **b** Proportion of eGenes discovered (FDR ≤ 1*%*) at age 70 (80) that validated (FDR ≤ 10*%*) at age 80 (70). The validation proportion of eGenes discovered at age 70 is significantly smaller than the proportion at age 80 (binomial proportion test; $\pi ^{val}_{70}-\pi ^{val}_{80} = -2.7\%, P = 3.3 \times 10^{-3}$). **c** Difference in expression *cis*-heritability between ages. The decrease in average *cis*-heritability with age is statistically significant (Wilcoxon signed rank test; $median(h^{2}_{80}-h^{2}_{70})= -1.4\%$, *P*=5.56×10^−14^). **d** Scatter plot of eQTL effect sizes at each age for genes that are eGenes in at least one age group. We observed a strong correlation of the fixed effect sizes between the two ages (Spearman’s *ρ*_*β*_=0.97). Blue line represents linear regression fit

We evaluated the association between expression of each autosomal gene and common *cis* genetic variants (within 1 Mb of the transcription start site) in each age group using linear regression models (“Materials and methods” section; Additional file 8). To minimize the impact of difference in statistical power between the two ages, we found and corrected for the number of hidden factors that maximized eQTL discovery separately in each age group (Fig. 3a and Additional file 1: Figure S7). After background noise correction, we detected 1326 eGenes, i.e., genes with at least one significant eQTL, at age 70 (8.5% of tested genes, FDR ≤ 1*%*) and 1264 eGenes at age 80. The depletion of eGenes at age 80, relative to age 70, was statistically significant (exact McNemar’s test; OR = 0.81, *P*=5.8×10^−3^).

Gene expression changes in response to environmental exposures. To address the question of how stable eQTL effects are with age, we estimated the rate of replication of discovery eGenes from one age group in the other age group (Fig. 3b). We observed a high proportion of eGenes that replicated for each age, 92.2% (94.9%) of eGenes discovered at age 70 (80) replicated at age 80 (70) (FDR ≤ 10*%*), although the rate of replication was lower for eGenes discovered at age 70, compared to eGenes discovered at age 80 (binomial proportion test; $\pi ^{val}_{70}-\pi ^{val}_{80} = -2.7\%,\ P= 3.3 \times 10^{-3}$). We used of a liberal FDR threshold for validation (FDR ≤ 10*%*) to ensure a low proportion of false age-specific eGenes, i.e., genes that do not validate at the second age. Results remained the same for a range of discovery and replication FDR thresholds, as well as minor allele frequency thresholds (Additional file 1: Figure S7B).

Gene expression may be influenced by both genetic and environmental factors. To address the question of the relative contribution of genetic variation, we estimated the *cis*-heritability for each gene in each age group using bi-variate linear mixed models (“Materials and methods” section; Fig. 3c). Consistent with results above, we found a small but statistically significant decrease in average *cis*-heritability with age (Wilcoxon signed rank test; $median(h^{2}_{80}-h^{2}_{70})= -1.4\%,\ P=5.56\times 10^{-14}$); the average heritability decreased from 18% at age 70 to 17% at age 80. We also estimated the genetic correlation of expression between the two ages, i.e., the proportion of expression variance shared between ages due to genetic causes. We observed a high genetic correlation of expression (*ρ*_*G*_=0.96; median across genes) and a strong correlation of the fixed effect sizes between the two ages (Fig. 3d); *ρ*_*β*_=0.70 and 0.97 transcriptome-wide and for genes that were eGenes in at least one age group, respectively.

There are two simple explanations for the depletion of eGenes at age 80, the lower replication rate at age 70, and the small decrease in *cis*-heritability with age: first, that the influence of genetic variation is reducing with age, or second, that the influence of environmental perturbations is increasing with age. The latter explanation is supported by the strong correlation of genetic effects as well as the lack of eQTLs for the change in gene expression with age (data not shown). Together, these findings suggest that genetic effects on gene expression are largely stable late in life and that environmental perturbation is gradually increasing with age, leading to lower SNP heritability estimates and lower power to detect eQTL as age increases.

We contrasted the eGenes with the genes that showed DE by age; 7.2% of the genes with eQTLs at both ages and 9.7% of the genes with eQTLs only at age 70 showed significant differences in expression by age. Last, only 2% of the genes that showed loss in genetic regulation with age are signature genes with cell-type-specific expression, indicating that our observations are very unlikely to be driven by differences in cell-type composition with age.

### Allele-specific expression by age across the transcriptome

**Fig. 4:**
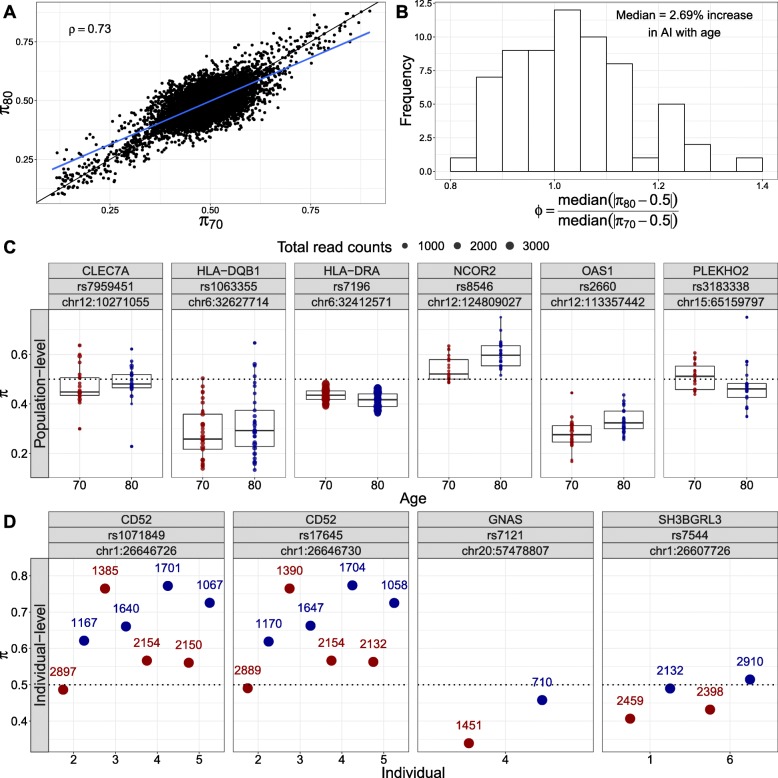
Population- and individual-level allele-specific expression across the genome. **a** Reference allele proportions (*π*) at the two ages of each individual are largely consistent (Spearman’s *ρ*=0.73, across individuals and sites). Blue line shows linear regression fit. **b** Global allelic imbalance increases with age by 2.69% (median across individuals and sites; Wilcoxon signed rank test; *P*=1.6×10^−2^). Distribution of median allelic imbalance *ϕ*=|*π*−0.50|) ratio of the two samples of each individual across all heterozygous sites. **c** Sites with significant local population-level differential ASE with age effects. Six sites show significant differential ASE with age effects. **d** Six individuals on three genes, i.e., *CD52*, *GNAS*, and *SH3BGRL3*, and four sites show individual-level differential ASE with age (FDR ≤ 5*%*). Points indicate reference allele proportion for each individual and site; color indicates age. Numbers on top of points show the total number of reads supporting the site at each age

We investigated transcriptome-wide patterns of allele-specific expression (ASE) with age. At the global level, reference allele proportions within an individual were largely consistent (Spearman’s *ρ*=0.73; Fig. 4a). Moreover, we observed a 2.6% increase in allelic imbalance (AI) with age (median across individuals and sites; Wilcoxon signed rank test; *P*=1.6×10^−2^; Fig. 4b). At the local level, as with total expression, we focused on both population- and individual-level differences in ASE with age. The former analysis requires the sites to be heterozygous across multiple individuals and captures, among others, age-interacting *cis*-regulatory effects while the latter captures effects of rare/personal variants or somatic mutations, e.g., as a result of clonal hematopoiesis.

At the population level, sites from six genes showed significant differential ASE with age (FDR ≤ 5*%*; Fig. 4c). *HLA-DRA*, *NCOR2*, and *PLEKHO2* show a significant gain of AI with age (likelihood ratio test (LRL); *P*=9.7×10^−6^, 4.1×10^−5^, and 4.2×10^−5^, respectively) while *CLEC7A*, *OAS1*, and *HLA-DQB1* show a significant loss of AI with age (LRT; *P*=7.7×10^−6^, 4.1×10^−5^, and 3.9×10^−6^, respectively). Most of these genes are involved in the immune system and have been previously implicated in the aging process [42, 43]. Most notably, *NCOR2* expression and its occupancy on peroxisome proliferator-activated receptor (PPAR) target gene promoters are increased with age in major metabolic tissues. Shifting its repressive activity towards PPARs, by selectively disabling one of its two major receptor-interacting domains, resulted in premature aging in mice and related metabolic diseases accompanied by reduced mitochondrial function and antioxidant gene expression [44]. *CLEC7A* is the only gene with differential ASE that is also significantly downregulated with age and a signature gene with macrophage-specific expression.

At the individual level, six individuals show differential ASE with age in four sites from three genes (FDR < 5*%*; Fig. 4d). *GNAS*, which showed a nominally significant loss in population-level AI with age (LRT *P*=3.4×10^−3^), also showed a significant decrease in individual-level AI with age. Moreover, two individuals showed a significant loss in AI with age for *SH3BGRL3*, a gene whose expression mean has been shown to decrease with age in the human skin [9] and whose expression variance has been shown to increase with age in rat retina [45]. Last, three individuals showed a significant gain in AI with age for two exonic SNPs in *CD52* while one individual showed loss of AI. None of these genes showed significant DE with age or are known to have cell-type-specific expression.

### Age-specific alternative splicing across the transcriptome

**Fig. 5:**
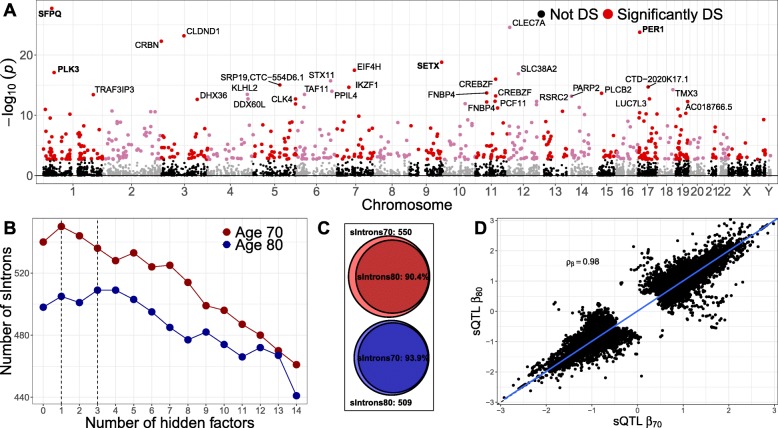
Population-level age-specific splicing across the transcriptome. **a** Manhattan plot of the splicing-age discoveries. Each dot represents a cluster in a gene; the *x*-axis gives the position of the cluster in the genome, and the *y*-axis represents the strength of the association with age. We find 294 age-associated genes with 503 clusters of alternatively excised introns (3.4% of tested genes and clusters; FDR ≤ 5*%*). Three of the top ten genes that had the strongest association with age, i.e., *SFPQ*, *PER1*, and *SETX*, are related to the circadian rhythm, disruption of which accelerates aging [46]. *PLK3*, which was also in the top ten most associated genes, is implicated in stress responses and double-strand break repair. **b** Number of introns with at least one significant sQTL (sIntrons) at age 70 and 80 for uncorrected analysis (number of hidden factors = 0) and analysis corrected for one up to 14 hidden factors. We detected 550 and 509 sIntrons at age 70 and 80 (FDR ≤ 5*%*), respectively. The depletion of sIntrons at age 80, relative to age 70, is statistically significant (exact McNemar’s test; *P*=8.6×10^−3^). Dashed line indicates the number of hidden factors that maximizes discovery at each age. **c** Proportion of sIntrons discovered at age 70 (80) that validated (FDR ≤ 20*%*) at age 80 (70). The validation proportion of sIntrons at age 70 is significantly smaller than the proportion at age 80 (binomial proportion test; *P*=2.2×10^−2^), indicating a decrease in genetic regulation with age. **d** Scatter plot of sQTL effect sizes at each age for introns that are sIntrons in at least one age group. We observed a strong correlation of the fixed effect sizes between the two ages (Spearman’s *ρ*_*β*_=0.98). Blue line represents linear regression fit

We investigated associations of transcriptome-wide changes in alternative splicing with age and discovered 503 clusters of alternatively excised introns from 294 genes (“Materials and methods” section) whose splicing levels were significantly associated with age (FDR ≤ 5*%*, Fig. 5a, Additional file 9); 11% of these genes showed also significant DE with age. GO enrichment analysis showed significant enrichment (FDR ≤ 5*%*) of terms related to regulation of RNA splicing, apoptosis, and leukocyte differentiation (Additional file 10). The strongest associations with age were found for genes related to the circadian rhythm (Fig. 5a), disruption of which accelerates aging [46], i.e., *SFPQ*, *PER1*, and *SETX*. *SFPQ* regulates the circadian clock by repressing the transcriptional activator activity of the *CLOCK-ARNTL* heterodimer and plays a role in the regulation of DNA virus-mediated innate immune response. Intron retention and nuclear loss of *SFPQ* are molecular hallmarks of amyotrophic lateral sclerosis (ALS) [47]. *PER1* is a member of the Period family of genes and is expressed in a circadian pattern in the suprachiasmatic nucleus, the primary circadian pacemaker in the mammalian brain. Genes in this family encode components of the circadian rhythms of locomotor activity, metabolism, and behavior. This gene is upregulated by *CLOCK-ARNTL* heterodimers, but then represses this upregulation in a feedback loop using PER/CRY heterodimers to interact with *CLOCK-ARNTL*. *SETX* is implicated in transcription termination and DNA double-strand breaks damage response generated by oxidative stress [48]. Mutations in this gene have been associated with juvenile ALS [49]. *SETX* is also required for the transcriptional termination of PER1 and CRY2, thus playing an important role in the circadian rhythm regulation.

### Stability of genetic regulation of alternative splicing across the transcriptome with age

We tested for genetic variants that affect alternative gene splicing on the autosomes at each age. For each gene, we quantified intron usages with LeafCutter [50] and evaluated the association between intron usage ratios at each age and genetic variants within 100 kb of the intron (“Materials and methods” section). After correction for background noise (Fig. 5b), we detected significant sQTLs for 550 introns at age 70 and 509 introns at age 80 (1.4% of tested introns, FDR ≤ 5*%*, Additional file 11). The depletion of introns with at least one significant sQTL (sIntrons) at age 80, relative to age 70, was statistically significant (exact McNemar’s test; *P*=8.6×10^−3^).

To study the stability of sQTL effects with age, we estimated the rate of replication of discovery sIntrons from one age group in the other age group (Fig. 5c). While we observed a high replication proportion at both ages, 90.4% (93.9%) of sIntrons discovered at age 70 (80) replicated at age 80 (70) (FDR ≤ 20*%*), the replication rate for sIntrons discovered at age 70 was significantly smaller, compared to sIntrons discovered at age 80 (binomial proportion test; *P*=2.2×10^−2^).

Last, we observed a strong correlation of sQTL effect sizes between the two ages (Fig. 5d, Spearman’s *ρ*_*β*_=0.98) as well as a lack of sQTLs for the change in alternative splicing with age (data not shown). This suggests that, similar to gene expression, genetic effects on alternative splicing are largely stable late in life and that increase in environmental noise underlies the loss of sQTLs as individuals age.

## Discussion

We have studied the combined effects of age and genetics on gene expression and alternative splicing in 65 humans from the general population sampled twice 10 years apart. Our focus on 70- and 80-year-old elderly individuals was designed to capture transcriptome changes during a period of high morbidity and mortality; the average life expectancy in Sweden is 80 and 83 years for men and women, respectively. We observed that individuals were more similar to their own gene expression levels between the two ages than to other individuals of the same age. This indicates that a larger proportion of gene expression variance is explained by shared genetics and environment than by the advanced aging process.

Despite the relative stability of expression profiles within individuals over time, we were able to identify 1291 genes with significant changes in expression. Pathways related to the adaptive immune system, cell signaling, and inflammatory response were among those enriched for downregulated genes while upregulated genes were enriched for pathways related to oxidative phosphorylation, adaptive immune system, and metabolism of proteins. Many of these functions have been previously described as hallmarks that represent common denominators of aging [51]. Moreover, 18 of the differentially expressed genes are previously known to be complex trait-associated genes where gene expression levels modulate risk.

Because the rate of aging varies among individuals, humans become increasingly different from each other with age [52]. Thus, chronological age fails to provide an accurate indicator of the aging process. Longitudinal studies offer a better understanding of the aging process by studying the same individuals throughout their lifespan. By collecting serial assessments, individuals can be compared to their own measurements at different time points rather than individuals of different ages from different environments. Recent longitudinal studies have begun to highlight the importance of individual-level molecular profiling to identify important health factors [53, 54]. Using our longitudinal design, we were able to analyze individual changes in gene expression and identified 529 individually dynamic genes with functions related to regulation of proteolysis and immune response. The sharing of immune-related function for both population and individual differentially expressed genes may, in part, be explained by observations of increased immune dysregulation and transcriptional variability with age [55, 56].

Genetic regulation of gene expression is involved in the etiology of many complex human traits [57, 58]. Previous studies in model organisms have reported a reduction in these associations with age [5]; however, less is known about the extent of genetic dysregulation with age in humans. Cross-sectional studies in humans have identified age-specific eQTLs for three [8] and ten [10] genes, respectively. A smaller 2-year longitudinal study of middle-aged females found two genes with time-dependent associations [22]. Our results indicate a small but significant global reduction in genetic control and gene expression heritability with age. This reduction could be due to several factors, such as the diminution of the level of expression of transcription factors, epigenetic modification, or genomic instability. Notably, while aging led to a reduction in genetic control, we observed an increase in the levels of allele-specific expression with age. The high correlation of genetic effects and the increase in allelic imbalance with age suggests that increasing environmental variance as opposed to decreased genetic variance underlies a component of the reduction in heritability and loss of genetic effects.

Deregulation of precursor mRNA splicing is associated with many illnesses and has been linked to age-related chronic diseases. There are no prior longitudinal studies of the human transcriptome [22, 23] assessing the dynamics of alternative splicing and its genetics. We found 294 genes, related, among others, to regulation of RNA splicing and apoptosis, with age-specific alternative splicing. Three of the top ten genes with the strongest association of alternative splicing with age are related to the circadian rhythm, disruption of which is known to accelerate aging [46]. In addition to changes in alternative splicing with age, we also observed a reduction in the number of genetic associations with splicing between the two ages highlighting similar patterns of dysregulation for both expression and splicing.

In summary, we present the first long-term, longitudinal characterization of expression and splicing changes as a function of age and genetics. Our findings indicate that, although gene expression and alternative splicing and their genetic regulation are mostly stable late in life, a small subset of genes is dynamic and is characterized by changes in expression and splicing and a reduction in genetic regulation, most likely due to an increase of environmental variance.

## Materials and methods

### Study cohort

The PIVUS study is a population-based study of the cardiovascular health in the elderly [28]. The PIVUS cohort is comprised of 1016 individuals (509 females and 507 males) of Swedish ancestry living in Uppsala, Sweden, from 2001 to 2005. Participants were examined at age 70 and 80 with deep phenotyping, and blood was frozen immediately upon collection. Our focus on 70- and 80-year-old elderly individuals was designed to capture changes during a period of high morbidity and mortality; the average life expectancy in Sweden is 80 and 83 years for men and women, respectively. A detailed description of the recruitment and phenotype data for this cohort is provided elsewhere [28].

### RNA isolation and sequencing

Gene expression was quantified for 65 individuals (35 females; 30 males) at both ages (130 samples). The RNA extraction, library preparation, and sequencing for the two samples from each individual was always performed in pairs and by the same technician to minimize confounding effects. Total RNA was extracted from 400 *μ*L whole blood using the NucleoSpin RNA Blood Kit (Macherey-Nagel, Düren, Germany) according to the manufacturer’s directions. Samples were eluted in 60 *μ*L RNase-free H_2_O. A small aliquot of each sample was set aside for quality assessment, and the remainder was immediately stored at − 80 ^∘^C. The RNA yield was estimated by measuring absorbance at 260 nm on the Nanodrop 2000 (Thermo Fisher), and RNA purity was determined by calculating 260/280 nm and 260/230 nm absorbance ratios. RNA integrity was assessed on the Agilent Bioanalyzer using the RNA 6000 Nano Chip kit (Agilent Technologies). An RNA integrity number (RIN) was assigned to each sample by the accompanying Bioanalyzer Expert 2100 software.

cDNA libraries were constructed following the Illumina TrueSeq Stranded mRNA Sample Prep Kit protocol and dual indexed. The average size and quality of each cDNA library were determined by the Agilent Bioanalyzer 2100, and concentrations were determined by Qubit for proper dilutions and balancing across samples. Twenty pooled samples with individual indices were run on an Illumina NextSeq 500 (high-output cartridge) as 2 × 75 paired end sequencing. Output BCL files were FASTQ-converted and demultiplexed.

### Genotyping and imputation

DNA was extracted and genotyped on the Illumina OmniExpress and Cardio-Metabochip arrays for more than 700K SNPs. Genotype data quality control was described elsewhere [59]. Only 63 of the 65 RNA-sequenced individuals passed genotype quality control; thus, all analyses involving genotype data, e.g., eQTL analysis, were performed on these 63 individuals with complete data. Genotype data was phased using SHAPEIT [60] and imputed with Impute2 (v2.3.2) [61] using the CEU haplotypes from the 1000 Genomes Project Phase-3 reference panel [62]. Post imputation quality control is described in Additional file 1.

### RNA-Seq quality control

Picard, Samtools [63], and other metrics were used to evaluate data quality (Additional file 1: Figure S1). Only genes that passed expression threshold were used; genes were considered expressed if, at both ages, they had, on average, at least 5 counts and 0 counts in no more than 20% of individuals (to minimize tails). A total of 16,086 genes were considered expressed. Gene expression data was library-size-corrected, variance-stabilized, and log2-transformed using the R package DESeq2 [64]. We refer to this version of the data as “raw data” as it is not corrected for global determinants of gene expression variability (see below).

### Background noise correction of gene expression data

It is well known that major components of expression variability can often be attributed to technical factors, e.g., RNA library size, that introduce unwanted, systematic variability in the data [29, 30]. Recovering and correcting for such factors can improve power to find differentially expressed/spliced genes or identify e/sQTLs. When these factors are correlated to the variable of interest, in our case age, they can act as confounders and correcting for such factors is necessary in order to avoid bias and prevent false discoveries.

To identify potential measured confounders, we search for measured factors that are associated both with age and gene expression. RIN and RNA concentration are moderately correlated with age (Spearman’s *ρ*= − 0.46 and − 0.30, Additional file 1: Figure S3), and they are both associated with gene expression (*π*_1_ = 61.43% and 22.19%, Additional file 1: Table S1). To extract unmeasured components of gene expression variability (referred to as hidden factors), we used surrogate variable analysis (SVA) as implemented in the R package smartSVA [65]. We set age as variable of interest and allowed for the factors to be correlated to age (Additional file 1: Figure S3). While the latter could reduce our power to identify DE genes, when correcting our analysis for these factors, it is a necessary step to avoid false-positive associations. More details about the implementation of SVA can be found in Additional file 1. We selected SVA, as opposed to other background noise correction methods [30, 66], on the basis of recent work that shows it is robust to spurious associations when setting a variable of interest [67]. Using the Buja and Eyuboglu method [68], we estimated the number of hidden factors that explains a significant amount of the expression variability to be 15.

All results in the main paper are corrected for hidden factors extracted by SVA as well as RIN and RNA concentration, to avoid false positives. In Additional file 1, we show results from uncorrected analyses and analyses corrected for measured factors (Additional file 1: Table S1), or hidden factors extracted by SVA without setting age as a variable of interest (Additional file 1: Figure S5A).

### Hierarchical clustering of gene expression

We performed hierarchical cluster analysis on the sample-to-sample distance matrix of the expression data. To compute the sample-to-sample distance matrix, we used the R function hclust from the stats package. We used the Euclidean distance measure to determine the distance between sets of observations. We used the *complete linkage* clustering strategy, a method that aims to find similar clusters. Samples were classified as “clustered with individual ID” if their nearest neighbor based on the dendrogram was their own sample from another age.

### Differential expression by age analysis

For each gene, we fit the following linear mixed model: expression ∼ individual (random) + age (fixed) + 15 hidden factors (fixed) + RIN (fixed) + RNA concentration (fixed), using the lme4 R package [69]. Age was coded as 0 and 1 for individuals at age 70 and 80, respectively. *P* values were calculated based on Satterthwaite’s approximations implemented in the lmerTest R package [70]. Significance of the results was assessed using the Storey and Tibshirani [71] *q*-value method implemented in the qvalue R package to control the FDR at 5%.

### Enrichment analysis for differentially expressed genes

Pathway enrichment analyses for DE genes were performed using GSEA [72], a computational method that determines whether an a priori defined set of genes shows statistically significant, concordant differences between two biological states (here age 70 vs 80). Resulting *P* values are adjusted for multiple testing using the qvalue method [71] controlling FDR at 5%.

### Replication of differential expression results in other blood studies

We assess the significance of the overlap between our top 1000 DE genes from PIVUS and the top 1000 DE gene in CHARGE [11] and SardiNIA [36] using the exact test of multi-set intersection implemented in the R package SuperExactTest [73]. We perform GO enrichment analysis for intersect genes that are shared between the three studies using WebGestaltR [74].

### Identification of individuals with outlying age trajectories

We only looked for outliers on autosomal genes and among the 61 individuals that cluster with their own sample at another age (Fig. 1a). Individuals are outliers for a gene, if their change in the expression of the gene between the two ages (*E*_80_−*E*_70_) falls outside the (*Q*_1_−3×*IQR*,*Q*_3_+3×*IQR*) range, where *Q*_1_ and *Q*_3_ are the 25th and 75th percentiles and IQR is the interquartile range of the distribution.

### eQTL mapping

We mapped eQTLs at each age using the linear regression models implemented in the MatrixEQTL R package [75]. For the analysis correcting for measured/hidden factors, we include the factors as covariates. To call eGenes at each age, we performed multiple testing at the gene and eQTL level using the hierarchical testing procedure implemented in the R package TreeQTL [76] controlling the FDR at 1%, both at the gene and gene-SNP level. The *P* values for the level 1 hypotheses (eGenes) were computed using Simes’ rule [77] on the families they index (collection of eQTL *P*values for each gene). This summary of the evidence for the global null hypothesis is relatively robust to dependence [78]. The final number and list of eGenes at each age was obtained using the number of expression hidden factors that maximized discovery at each age, i.e., 10 hidden factors (Fig. 3a).

### Stability of eQTL effects with age

We estimated the rate of replication of discovery eGenes from one age group in the other age group using a two-step FDR approach from validation theory [79]. Specifically, we first discover eGenes at age 70 at 1% FDR, as described above, and then we validate them at age 80 at 10% and 20% FDR, by performing eQTL mapping only for these genes.We reverse the process for age 80.

### Heritability of gene expression at each age

For each gene, we used the bivariate GREML method, implemented in the GCTA software [80], to estimate the *cis*-heritability of expression at each age as well as the genetic correlation of expression between the two ages. To estimate the average *cis*-heritability with age, we use the beta regression models implemented in the R package betareg [81], modeling the logit of the *cis*-heritability of each gene as a function of age, i.e., *logit*(*h*^2^)=*α*+*β*×*age*, where *h*^2^ is the estimated *cis*-heritability of a gene, *β* is the effect of age on heritability, and *age* is coded as above. Then, the estimated average *cis*-heritability at age 70 and 80 is given by $h^{2}_{70} = 1/\left (1+ \exp (-\alpha)\right)$ and $h^{2}_{80} = 1/\left (1+ \exp (-(\alpha +\beta))\right)$, respectively. When testing for the significance of the difference in *cis*-heritability with age, we also adjust for the standard error of the heritability, in order to take into account cases where the estimate of the heritability at one of the two ages is noisier.

### Quantifying allele-specific count data from RNA-Seq data

Read mapping bias was removed by following the WASP pipeline [82]. The GATK tool ASEReadCounter was used to count reads at exonic heterozygous sites. Only bi-allelic SNPs with reference allele proportion ≥.1 or ≤.9 were considered. Correlation of reference allele proportions between the two ages and testing for changes in global AI and local individual-level differential ASE with age was performed using only sites supported by at least 100 and 50 reads at each age of an individual, respectively. The local population-level differential ASE with age analysis was performed on sites supported by at least 20 reads at each age of an individual in at least 20 individuals.

For the two individuals for which genotypes were not available, we called genotypes at heterozygous sites using the GATK RNA-Seq variant calling pipeline [83]. We limited the variants to only those that were found to be heterozygous in at least one of the other PIVUS samples. We then ran the WASP pipeline described above to obtain allelic read counts for these samples.

### Age-specific ASE mapping

Let *π*_*ijk*_ and *ϕ*_*ijk*_=|*π*_*ijk*_−0.5| denote the proportion of reads supporting the reference allele and the allelic imbalance, i.e., absolute deviation from allelic balance, for individual *i* (*i*=1,…,65), at age *j* (*j*=70,80), and heterozygous site *k* (*k*=1,…,*K*_*ij*_). Moreover, let *ϕ*_*ij*∗_ the median allelic imbalance for *i* at age *j* and $\phi _{i**} = \frac {\phi _{i80*}}{\phi _{i70*}}$ the ratio of median AI between the two ages of individual *i* across all sites. We test for a statistically significant increase in global AI with age (*H*_0_:*μ*≤1 vs *H*_1_:*μ*>1, where *μ* median of the *ϕ*_*i*∗∗_’s) using the one-sample Wilcoxon signed rank test.

At the local level, we test for population- and individual-level differences in ASE with age using the beta-binomial generalized linear (mixed) models implemented in EAGLE (v2.0) [84, 85]. In short, for each exonic SNP, we model the alternative allele count for heterozygous individual *i*, at age *t* using a beta-binomial distribution, i.e., *y*_*it*_∼*BB*(*n*_*it*_,*σ*(*ϕ*_*i*_*β*_*t*_),*c*), where *n*_*it*_ is the total number of reads for *i* in *t*, *σ*() is the logistic function, *ϕ*_*i*_ represents the phase between the causal *cis*-SNP and the exonic SNP and is treated as a latent variable taking values in {−1,+1}. We learn a prior *π*=*P*(*ϕ*_*i*_=+1) across all individuals and marginalize (sum) over the possible values of *π*. *β*_*t*_ is the effect if age on the reference allele proportion. Last, *c* is the concentration parameter which we learn per exonic SNP using maximum a posteriori probability estimation with a *Gamma*(1.0001,1×10^−4^) prior. For analyses at the population level (across multiple heterozygous individuals), we adapted EAGLE to handle repeated samples from the same individual, i.e., *y*_*it*_∼*BB*(*n*_*it*_,*σ*(*ϕ*_*i*_*β*_*t*_+*u*_*i*_),*c*), where *u*_*i*_∼*N*(0,*v*) is a per individual, per exonic SNP random effect. We use variational Bayes EM to approximately integrate over all *u*_*i* while optimizing with respect to the other parameters. EAGLE2 uses the Stan probabilistic programming language [86].

### Quantification of alternative splicing

To quantify alternative splicing events, we followed the LeafCutter [50] pipeline. In short, we first mapped the 130 RNA-Seq samples from PIVUS to the human genome (hg19) using STAR, allowing de novo splice junction predictions. We then used LeafCutter [50] to identify alternatively excised introns by pooling all junction reads. LeafCutter then defines “clusters” of alternatively excised introns that represent alternative splicing choices. This resulted in 78,373 alternatively excised introns from 24,126 clusters. Each cluster comprises of, on average, 3.2 introns (median = 3, min = 2, max = 51).

To identify alternative splicing events that are suitable for differential splicing and sQTL by age analysis, we first excluded clusters with more than 10 introns. Then, we restricted our analyses to active introns, i.e., introns that are supported by at least 10*%* of the total number of reads supporting the clusters they belong to in at least 25% of samples, considering each age separately. Clusters with less than two active introns after this step were filtered out. Last, we only considered clusters that exhibit some minimum splicing variability, i.e., clusters with Hellinger’s distance ≥ 1*%* [87]. In the end, we had 14,917 clusters and 36,713 introns.

### Background noise correction of alternative splicing data

As with the gene expression data, we used SVA [65], setting age as variable of interest, to identify major components of alternative splicing variability (Additional file 1: Figure S8). More details about the implementation of SVA can be found in Additional file 1. Using the Buja and Eyuboglu [68] method, we estimated the number of hidden factors that explains a significant amount of the alternative splicing variability to be 14.

### Differential splicing by age analysis

To identify alternative splicing events with age, we used the Dirichlet-multinomial generalized linear model implemented in LeafCutter [50]. We adjusted our analysis for measured factors that explained more than 0.5% splicing variability, i.e., RIN, proportion of intronic bases, median insert size, and extraction year (Additional file 1: Figure S8B). *P* values of association with age were calculated based on the likelihood ratio test. To call dsGenes, i.e., genes with at least one significantly DS cluster, as well as DS clusters for each dsGene, we used the R package TreeQTL [76] controlling the FDR at 5% both at the gene and gene-cluster level.

### sQTL mapping

We used linear regression, as implemented in MatrixEQTL [75], to test for associations between ratios of alternatively excised introns at each age group and variants within 100 kb of the intron clusters, adjusting for splicing hidden factors. We control the FDR at 5% both at the intron and intron-SNP level using TreeQTL [76].

## Supplementary information

**Additional file 1** Supplemental methods, tables, and figures.

**Additional file 2** Summary statistics for differential expression by age analysis.

**Additional file 3** Summary of results from gene set enrichment analysis for genes differentially expressed with age.

**Additional file 4** Summary of co-localization results of gWAS traits and gTEx whole-blood eQTL summary statistics for dE genes.

**Additional file 5** Genes differentially expressed by age in pIVUS, cHARGE, and Sardinia.

**Additional file 6** Genes differentially expressed by age in pIVUS that are known aging- and longevity-related genes from the human aging genomic resources (HAGR) genAge and longevityMap databases.

**Additional file 7** Summary statistics for age-trajectory outliers.

**Additional file 8** Summary statistics for the eQTL analyses at each age.

**Additional file 9** Cluster- and gene-level summary statistics for differential splicing by age analysis.

**Additional file 10** Gene-ontology enrichment analysis for genes differentially spliced with age. XLS 30KB

**Additional file 11** Summary statistics for the sQTL analyses at each age.

**Additional file 12** Review history.

## Data Availability

The data for this study can be found in EGA under accession code EGAD00001004965 [88].
