# Supplementary material for: Genetic regulation of gene expression and splicing during a 10-year period of human aging
Source: Genome Biol. 2019 Nov 4;20:230. doi: 10.1186/s13059-019-1840-y (PMC6827221; doi:10.1186/s13059-019-1840-y)
Supplement: Supplementary file 1 — Additional file 1 Supplemental methods, tables, and figures. [file 13059_2019_1840_MOESM1_ESM.pdf]

Genetic regulation of gene expression and splicing during a ten-year period of human aging

Supplemental methods, tables, and figures

SNP array data and imputation

Genotype data quality control was described elsewhere[1]. In summary, 949 individuals passed genotype quality control. Genotype phasing and imputation was performed in all 949 individuals that passed quality control. Post-imputation quality control was performed as follows. SNPs with an imputation info-score below 0.4, a HWE $P \leq 10^{-6}$, or a MAF $\leq$ 5% in the 63 individuals with measured RNA-Seq and methylation were excluded. In total, 7,037,776 SNPs passed post-imputation quality control.

Expression quantification and quality control

The quality of the raw reads was assessed using `FastQC` (v0.11.5). The adaptors were clipped using `cutadapt` (v1.8.1)[2] requiring at least three bases to match (`--min overlap 3`) and removing processed reads shorter than 20 bases (`--min length 20`). RNA-Seq reads were mapped to the NCBI v37 H. sapiens reference genome using `STAR` (v2.4.2a)[3]. Only uniquely aligned reads were used for downstream quantification and analysis. The percentage of reads marked as PCR duplicates was computed using `Picard`. For the differential expression and eQTL analysis, PCR duplicates were not filtered out, since it has been shown that computational removal of duplicates does not improve power or FDR in differential expression analyses[4], but the proportion of PCR duplicates used as a technical covariate in downstream analysis. For the differential ASE analysis, PCR duplicates were removed. Mapping statistics from the BAM files were acquired through *Samtools flagstat* (v1.2)[5]. The 5' and 3' coverage bias, duplication rate and insert sizes were assessed using `Picard` tools (v2.0.1). `HTSeq` was used to quantify gene expression[6].

Expression data on the sample level were first corrected for library size using the `DESeq2` R package[7]. Genes were excluded if they had less than 5 counts on average for either age groups, and zero counts in more than 20% of individuals (to minimize tails). A total of 16,087 genes were expressed. For the eQTL analysis, genes from the sex chromosomes as well as mitochondrial genes were also excluded, leaving 15,729 genes in the analysis.

To identify potential outlier individuals, we performed PCA (Figure S1). Samples that demonstrated extreme values in the first two principal components on the expression levels were removed (more than 3 standard deviations). No samples were excluded based on this measure. To identify low quality samples, we applied several quality metrics (Figure S1). Samples were removed if they had insufficient reads ($\leq$20M), poor mappability ($\leq$60%), and low correlation with other samples (D-statistic $\leq$ 0.85). We also checked for sample mix-ups by comparing agreement between true and RNA-Seq-inferred heterozygous SNPs

for all possible pairs of RNA-Seq and genotype data. No samples were excluded based on these metrics. Three individuals with low RNA integrity number (RIN ≤ 5) were marked. These individuals were not excluded since they do not seem to appear as outliers in the PCA plots (Figure S1C).

Measuring DNA methylation and quality control

Extracted DNA was bisulphite-converted using the Zymo bisulphite conversion kit and hybridized to the Infinium HumanMethylation450 BeadChip (450k array). Signal intensities were measured with the BeadChip scanner. The 130 samples (with available RNA-Seq data) were distributed across two periods of methylation data collection, eight 96-well plates (5 plates for the 70-year old samples and 3 plates for the 80-year old samples), and forty-one 12-sample chips (24 chips for the 70-year old samples and 17 chips for the 80-year old samples).

Quality control was performed using the R package `minfi` (version 1.20.0)[8]. Missing values were imputed by the k-nearest neighbor approach using the `impute` R package (version 1.48.0)[9]. Background correction and dye-bias normalization were done using `noob`[10] through `minfi`. Signal intensities were converted to DNA methylation β-values, i.e. the ratio of methylated probe intensity to total (methylated + un-methylated) probe intensity.

Inferring cell-type frequencies from whole blood

Whole blood is a heterogeneous mixture of cell types. Since gene expression and DNA methylation vary across different cell types, correlations between the phenotype of interest (e.g. age) and the cell type composition may lead to a large number of false discoveries. False discoveries due to cell type heterogeneity can be addressed by adding the cell proportions as covariates. Since cell counts were not available for our samples, we used computational methods to estimate their composition.

To estimate blood cell composition from gene expression data, we used CIBERSORT[11]. While this tool was designed from micro-array data, it has been shown to have reasonably robust cross-platform performance. To estimate blood cell composition from DNA methylation data, we used Houseman's reference-based method[12], including their provided reference data and signature CpG sites. This approach was used on our methylation data after adjustment with `noob`[10]. Results are shown in Figure S2A.

Methylation-based cell-type frequency estimates are closer to expected values for adults of similar age, compared to expression-based estimates. Moreover, while methylation-based estimates are mainly correlated with biological covariates, expression-based estimates are highly correlated with technical factors Figure S2B. For these reason, we use methylation-based estimates in downstream analyses. Methylation-based estimates of B and CD8 T cells, granulocyte, and monocytes showed a significant difference between the two ages (2-sample Wilcoxon test; $P = 0.018$, 0.019, 1.21×10^{-4}, and 9.29×10^{-3}, Figure S2C).

Background noise correction in RNA-Seq experiments

We consider analyses corrected for either measured and/or inferred determinants of gene expression variability. Below we describe the selection of the known factors and the inference of the inferred factors.

Measured determinants of gene expression variability in RNA-Seq experiments

We considered 24 measured variables as candidate components of RNA-Seq variability, listed in Table S1. In order to decide which of the variables affect gene expression, we performed a multiple linear mixed model regression on the expression of each gene using the `lme4` R package[13]. We used the π_1 statistic[14] to detect technical covariates affecting a large number of genes, i.e. $\pi_1 \geq 5\%$, and only consider those covariates in subsequent analyses. Table S1 also lists the median % of gene expression variance accounted for (VAF) by each measured variable, estimated using the R package `variancePartition`[15], as well as the proportion of genes each variable was associated with at 5% FDR.

Figure sfig:KnownAndInferredFactorsA and C show the correlation of the variables (that have $\pi_1 \leq 5\%$) with age and the proportion of gene expression variance they explain. Since age is moderately correlated with RIN (Spearman's $\rho = -0.46, P = 5.16 \times 10^{-8}$) and RNA concentration (Spearman's $\rho = -0.30, P = 4.21 \times 10^{-4}$) and RIN and RNA concentration are associated with gene expression, these variables could act as potential confounders and we thus include them in the model for differential expression analysis.

Inferred determinants of gene expression variability in RNA-Seq experiments

We used surrogate variable analysis (SVA) to infer hidden factors from the RNA-Seq data. Two different algorithms for extracting hidden factors were considered: the two-step SVA procedure[16] without setting any covariate of interest, implemented in the `sva` R package[17], and the IRW-SVA algorithm, setting age as the covariate of interest[18], implemented in the `SmartSVA` R package[19]. The later algorithm, to which we hereafter refer as supervised SVA, attempts to protect the effect of age by identifying a subset of genes that show strong association with the underlying sources of gene expression heterogeneity but no association with age, also referred to as *negative* or *empirical* control genes.

We use the Buja and Eyuboglu method[20], a permutation-based selection rule for the number-of-factors problem, to estimate the number of hidden factors that explain a significant proportion of gene expression variability, larger than what would be expected by chance. Using the SVA method, we find 12 and 15 factors when performing the unsupervised and supervised algorithms, respectively.

We found that the inferred factors summarize multiple correlated measured factors (Figure sfig:KnownAndInferredFactorsB) with significant contribution to variability in the RNA-sequencing data (Figure sfig:KnownAndInferredFactorsB). Generally, we observed that the top factors largely correspond to technical factors such as RNA extraction date, RIN scores and factors specific to RNA-seq such as percent duplicated reads and others obtained from the Picard metrics and to a much lesser extent to biological factors such as estimates of cell type frequencies.

Co-localization analysis for DE genes

For each DE gene, we obtained colocalization posterior probabilities (CLPP) between GWAS summary statistics of several complex traits and GTEx[21] whole blood from the *LocusCompare* database (`http://locuscompare.ml:3838/`). We defined any locus with CLPP ≥ 0.05 to have sufficient evidence for colocalization.

Differential expression analysis in the SardiNIA study

The SardiNIA study consists of 605 individuals (56% females, average age 57) from 195 families with measured RNA-Seq[22]. From a total of 19,646 genes expressed in SardiNIA, 14,847 of them are also expressed in PIVUS, using the same threshold for calling a gene expressed (see above). To account for the family structure, we perform the differential expression analysis using the pedigree-based linear mixed-models implemented in the `coxme` R package[23]. Specifically, let E_{ij} and Age_{ij} denote the gene expression and age for the jth member of family i, and $\mathbf{x_{ij}}$ a p-dimensional vector with known / inferred determinants of gene expression for $i = 1, \ldots, n$ and $j = 1, \ldots, n_i$. Here we correct the analysis for sex. In the mixed models framework, a set of family-specific random effects $\mathbf{b_i} = (\mathbf{b_{i1}}, \mathbf{b_{i2}}, \ldots, \mathbf{b_{in_i}})^{\mathbf{T}}$ is introduced to model the within family dependencies and E_{ij} is modeled conditional on the $n_i \times 1$ random-effects vector $\mathbf{b_i}$, and covariate information Age_{ij} and $\mathbf{x_{ij}}$ as

$$
\begin{aligned}
E_{ij} &= \beta_0 + b_{ij} + \beta_1 Age_{ij} + \mathbf{x_{ij}^T}\boldsymbol{\beta_2} + \epsilon_{\mathbf{ij}} \\
(\boldsymbol{\epsilon_i}, \boldsymbol{b_i}) &\sim N_{2n_i}\left(\mathbf{0}_{\boldsymbol{2n_i}}, \boldsymbol{\Sigma}_i\right) \\
\boldsymbol{\Sigma}_i &= \begin{bmatrix} \sigma_\epsilon^2 \mathbf{I_{n_i}} & \mathbf{0}_{\boldsymbol{2n_i}} \\ \mathbf{0}_{\boldsymbol{2n_i}} & \sigma_b^2 \mathbf{R_{n_i}} \end{bmatrix}
\end{aligned}
$$

Where β_0 is the intercept term, β_1 is the fixed effect of age, and $\boldsymbol{\beta_2}$ is the p-dimensional regression coefficients vector for the additional covariates. Moreover, $\mathbf{R_{n_i}}$ is the coefficient of relationships matrix with elements $r_{jk} = 2^{-d_{jk}}$ with d_{jk} denoting the distance between subjects j and k in the pedigree and σ_b^2 the genetic variance parameter. σ_ϵ^2 is the residual variance and $\mathbf{I_{n_i}}$ is an $n_i \times n_i$ identity matrix.

References

[1] M. S. Artigas, L. V. Wain, S. Miller, et al., Sixteen new lung function signals identified through 1000 Genomes Project reference panel imputation, Nature Communications 6 (2015) 8658.

[2] M. Martin, Cutadapt removes adapter sequences from high-throughput sequencing reads, EMBnet.journal 17 (2011) 10–12.

[3] A. Dobin, C. A. Davis, F. Schlesinger, et al., STAR: ultrafast universal RNA-seq aligner, Bioinformatics 29 (2013) 15–21.

[4] S. Parekh, C. Ziegenhain, B. Vieth, W. Enard, I. Hellmann, The impact of amplification on differential expression analyses by RNA-seq, Scientific Reports 6 (2016) 25533.

[5] H. Li, B. Handsaker, A. Wysoker, et al., The sequence alignment/map format and SAMtools, Bioinformatics 25 (2009) 2078–2079.

[6] S. Anders, P. T. Pyl, W. Huber, HTSeqa Python framework to work with high-throughput sequencing data, Bioinformatics 31 (2015) 166–169.

[7] M. I. Love, W. Huber, S. Anders, Moderated estimation of fold change and dispersion for RNA-seq data with DESeq2, Genome Biology 15 (2014).

[8] M. J. Aryee, A. E. Jaffe, H. Corrada-Bravo, et al., Minfi: a flexible and comprehensive bioconductor package for the analysis of infinium dna methylation microarrays, Bioinformatics 30 (2014) 1363–1369.

[9] T. Hastie, R. Tibshirani, B. Narasimhan, G. Chu, impute: Imputation for microarray data, 2016. R package version 1.48.0.

[10] T. J. Triche, Jr, D. J. Weisenberger, D. Van Den Berg, P. W. Laird, K. D. Siegmund, Low-level processing of illumina infinium dna methylation beadarrays, Nucleic Acids Research 41 (2013) e90.

[11] A. M. Newman, C. L. Liu, M. R. Green, et al., Robust enumeration of cell subsets from tissue expression profiles, Nature Methods 12 (2015) 453–457.

[12] E. A. Houseman, W. P. Accomando, D. C. Koestler, et al., DNA methylation arrays as surrogate measures of cell mixture distribution, BMC Bioinformatics 13 (2012) 86.

[13] D. Bates, M. Mächler, B. Bolker, S. Walker, Fitting linear mixed-effects models using lme4, Journal of Statistical Software 67 (2015) 1–48.

[14] J. D. Storey, R. Tibshirani, Statistical significance for genomewide studies, Proceedings of the National Academy of Sciences 100 (2003) 9440–9445.

[15] G. E. Hoffman, E. E. Schadt, variancepartition: Interpreting drivers of variation in complex gene expression studies, BMC Bioinformatics 17 (2016) 483.

[16] J. T. Leek, J. D. Storey, Capturing Heterogeneity in Gene Expression Studies by Surrogate Variable Analysis, PLOS Genetics 3 (2007) e161.

[17] J. T. Leek, svaseq: removing batch effects and other unwanted noise from sequencing data, Nucleic Acids Res 42 (2014) e161.

[18] J. T. Leek, J. D. Storey, A general framework for multiple testing dependence, Proceedings of the National Academy of Sciences 105 (2008) 18718–18723.

[19] J. Chen, E. Behnam, SmartSVA: Fast and Robust Surrogate Variable Analysis, 2017. R package version 0.1.3.

[20] A. Buja, N. Eyuboglu, Remarks on Parallel Analysis, Multivariate Behavioral Research 27 (1992) 509–540.

[21] The GTEx Consortium, Genetic effects on gene expression across human tissues, Nature 550 (2017) 204–213.

[22] M. Pala, Z. Zappala, M. Marongiu, et al., Population- and individual-specific regulatory variation in Sardinia, Nature Genetics 49 (2017) 700–707.

[23] T. M. Therneau, coxme: Mixed Effects Cox Models, 2015. R package version 2.2-5.

[24] A. C. Shaw, D. R. Goldstein, R. R. Montgomery, Age-dependent dysregulation of innate immunity, Nature Reviews. Immunology 13 (2013) 875–887.

[25] D. Vilchez, I. Saez, A. Dillin, The role of protein clearance mechanisms in organismal ageing and age-related diseases, Nature Communications 5 (2014) 5659.

[26] C. B. Peterson, M. Bogomolov, Y. Benjamini, C. Sabatti, Treeqtl: hierarchical error control for eqtl findings, Bioinformatics 32 (2016) 2556–2558.

Supplemental Tables

Table S1: **Measured covariates that can introduce variability in RNA-sequencing experiment.** Table lists technical factors directly obtained from Picard QC metrics (Type "T/Picard"), factors relating to sample preparation and storage (Type "SP"), methylation-based cell type frequencies (Type "MCTF"), and factors measured in the clinic (Type "C"). For subsequent analyses, we only consider factors that affect a large proportion of genes ($\hat{\pi}_1 > 5\%$). Table also shows median % of expression variance accounted for (VAF) by each factor and genes each factor was associated with at 5% FDR. $\hat{\pi}_1$, VAF, and the number of associations for each covariate were estimated via a multiple linear mixed model per gene correcting for all uncorrelated covariates.

ID	Description	Type	$\hat{\pi}_1$	Median VAF (%)	% associations
1	Extraction year	SP	77.59	5.54	59.51
2	% Intronic bases	T/Picard	72.59	2.06	53.05
3	RNA integrity number	SP	61.43	1.85	34.33
4	CD8 T cells	MCTF	60.72	2.08	31.30
5	CD4 T cells	MCTF	56.23	1.57	23.94
6	Median insert size	T/Picard	49.16	1.02	19.39
7	Leukocytes count	C	45.09	0.72	10.02
8	Monocytes	MCTF	29.60	0.35	1.93
9	NK cells	MCTF	28.80	0.39	0.89
10	SBP	C	22.88	0.38	0.00
11	RNA concentration	SP	22.19	0.41	1.46
12	Min insert size	T/Picard	18.85	0.21	0.00
13	Sex	C	14.99	0.42	0.00
14	B cells	MCTF	10.23	0.33	0.83
15	DBP	C	9.47	0.32	0.00
16	Fasting blood glucose	C	9.15	0.21	0.00
17	Albumin	C	6.76	0.27	0.00
18	BMI	C	6.07	0.29	0.00
19	Alanine aminotransferase	C	4.03	0.18	0.00
20	Any medications	C	0.17	0.18	0.00
21	Smoking	C	0.00	0.17	0.00
22	Alkalic phosphates	C	0.00	0.23	0.00
23	Calcium	C	0.00	0.16	0.00
24	C-reactive protein	C	0.00	0.16	0.00

Supplemental Figures

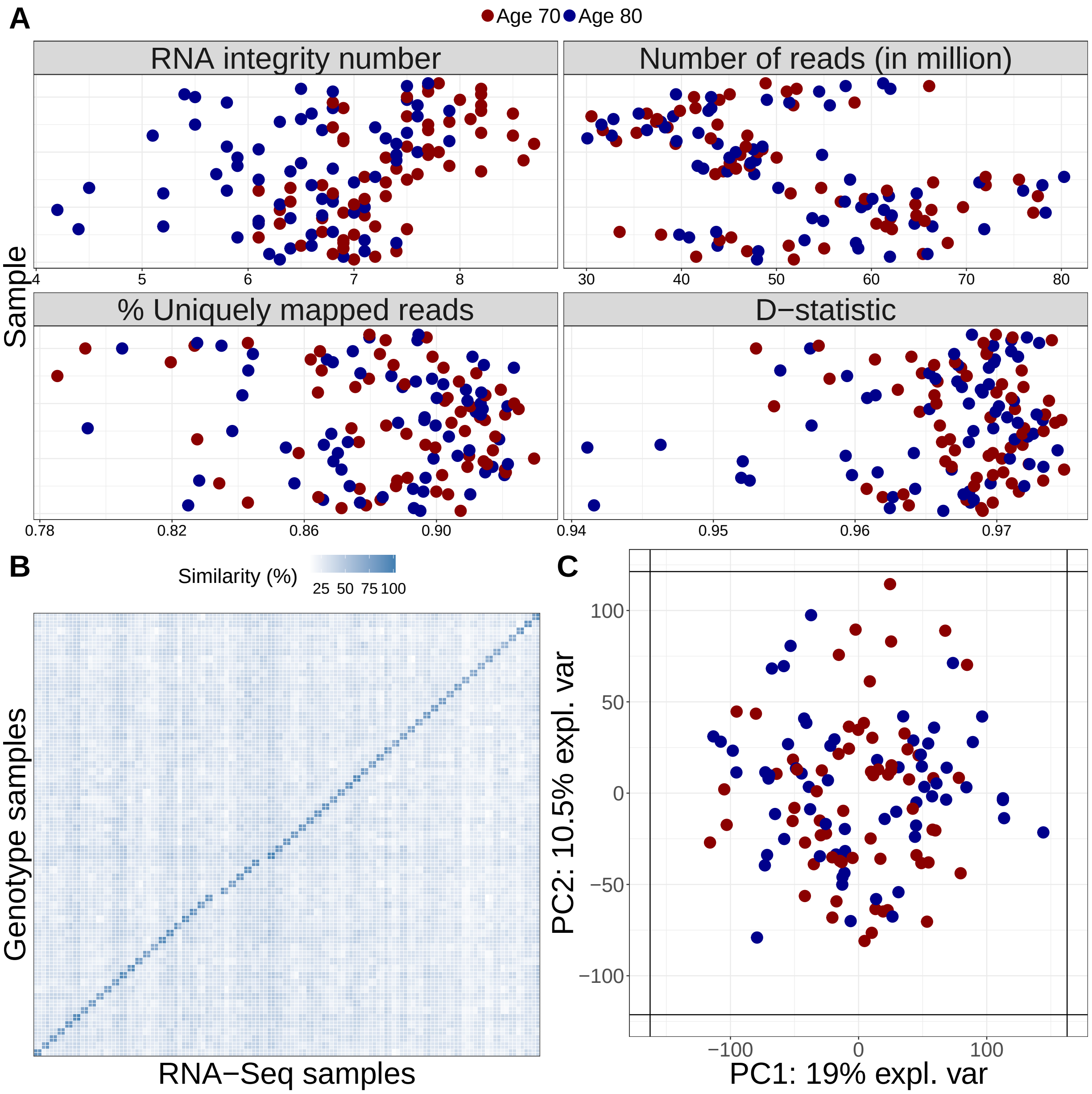

Figure S1: **RNA-Seq data quality control**. **(A)** Distribution of RNA integrity number, number of sequenced reads (in million), percent of uniquely mapped reads, and D-statistic across samples. The median RNA integrity number across samples was 6.9. All samples had at least 30M reads, at least 80% of their reads mapped uniquely, and their median Spearman expression correlation (D-statistic) with other samples was at least 0.9. **(B)** Concordance between SNP array and RNA-Seq called heterozygous loci. All RNA-seq samples are most similar to their own genotype sample (darker numbers in the diagonal). The four light colors in the diagonals refer to the two individuals (four samples) for which SNP genotypes were not available; these individuals are not similar to the genotype of any other sample. **(C)** Principal component analysis on expression data. No outliers are present based on the two first principal components (PC).

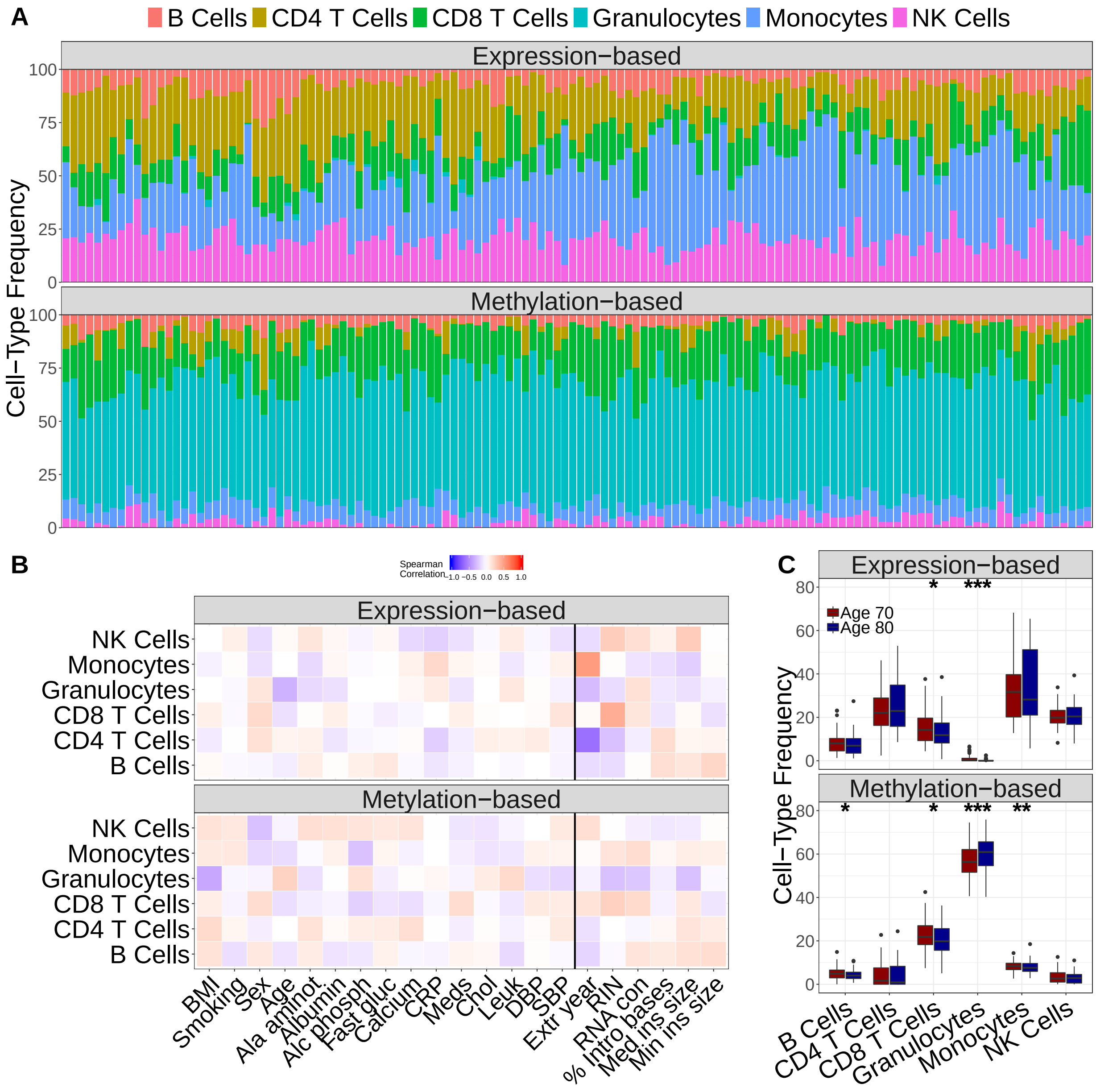

Figure S2: **Cell-type deconvolution in whole blood**. **(A)** Cell-type frequency estimates (in %). Each column contains relative estimates for each sample. Pairs of consecutive columns are 70- and 80-years-old samples of the same individual. Expression-based estimates are not close to expected values for adults of similar age. **(B)** Correlation of cell-type frequencies with biological and technical covariates (separated by black line). Expression-based estimates are highly correlated with technical covariates while methylation-based estimates are correlated with biological covariates. **(C)** Distribution of estimated cell-type frequencies by age. For expression-based estimates, we see a significant difference with ages for CD4 and CD8 T cells and granulocytes (2-sample Wilcoxon test; $P = 0.053$, .029, and 6.73×10^{-5}). For methylation-based estimates, B and CD8 T cells, granulocytes, and monocytes showed a significant difference between the two ages ($P = 0.018$, 0.019, 1.21×10^{-4}, and 9.29×10^{-3}). Significance codes: $'***' \leq 0.001$, $'**' \leq 0.01$, $'*' \leq 0.05$.

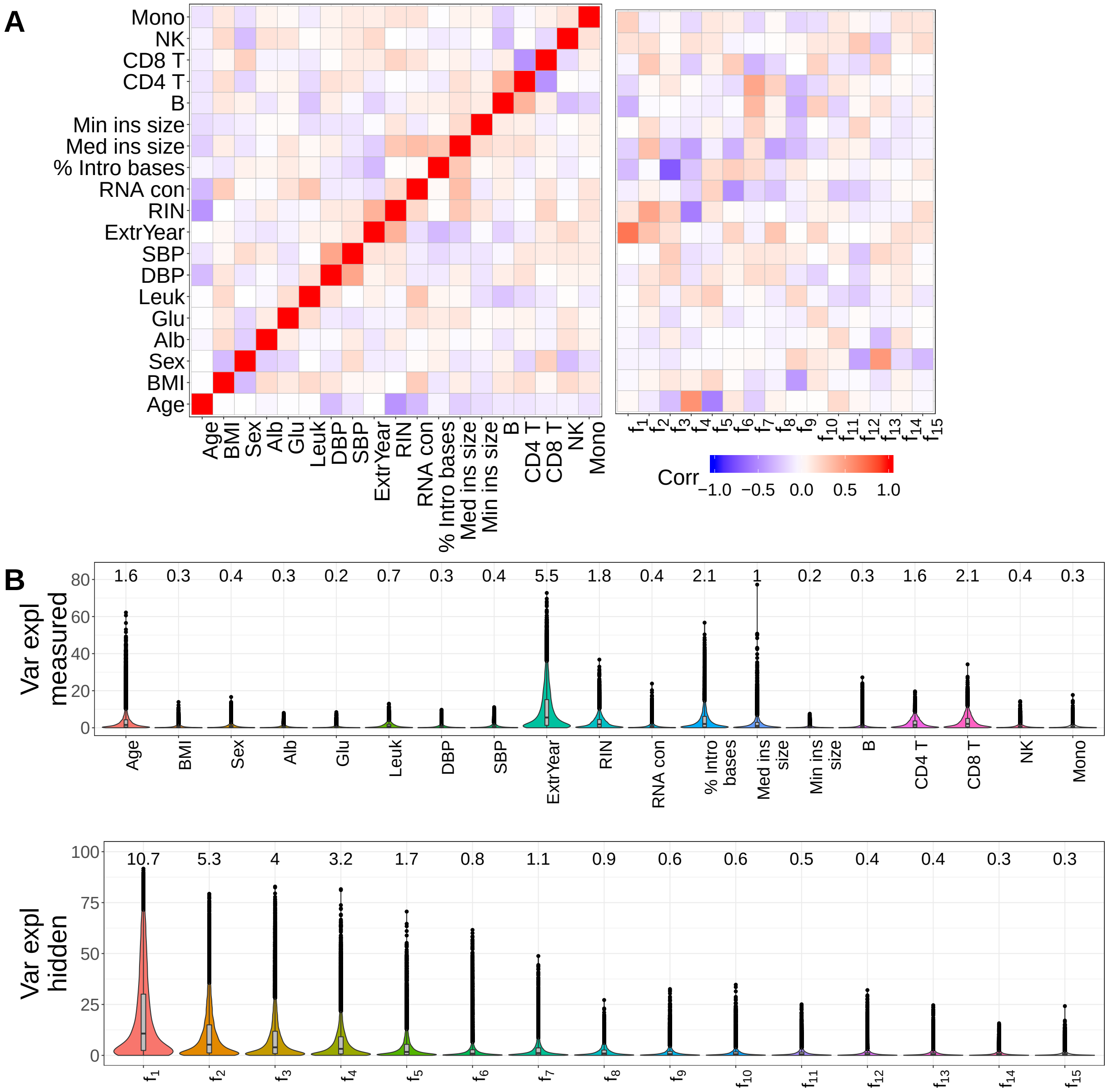

Figure S3: **Known and inferred determinants of gene expression variability**. **(A)** Correlation between measured covariates and with hidden factors. Age is moderately correlated with RIN (Spearman's $\rho = -0.46$, $P = 5.16 \times 10^{-8}$) and RNA concentration (Spearman's $\rho = -0.30$, $P = 4.21 \times 10^{-4}$). Hidden factors are correlated with several measured factors, e.g. Spearman's $\rho = .69$ between extraction year and first hidden factor. The fourth and fifth hidden factors are moderately correlated with age (Spearman's $\rho_{f_4,age} = 0.56$ and $\rho_{f_5,age} = -0.55$). The fourth hidden factor is also correlated with RIN (Spearman's $\rho = 0.56$), consistent with the fact that age and RIN are moderately correlated. **(B)** Proportion of gene expression variance explained (VE) by measured and hidden factors. VE for each measured factor is estimated by fitting a multiple linear mixed model with all measured factors and age for each gene. Consistent with results in (A), extraction year accounts for the largest proportion of gene expression variance (VE mean= 9.89%, median = 5.54%). Measured covariates are listed in the same order as Table S1. Moreover, the first hidden factor explains about 10% of gene expression variance (median across genes) while the 15th one explains about .3%.

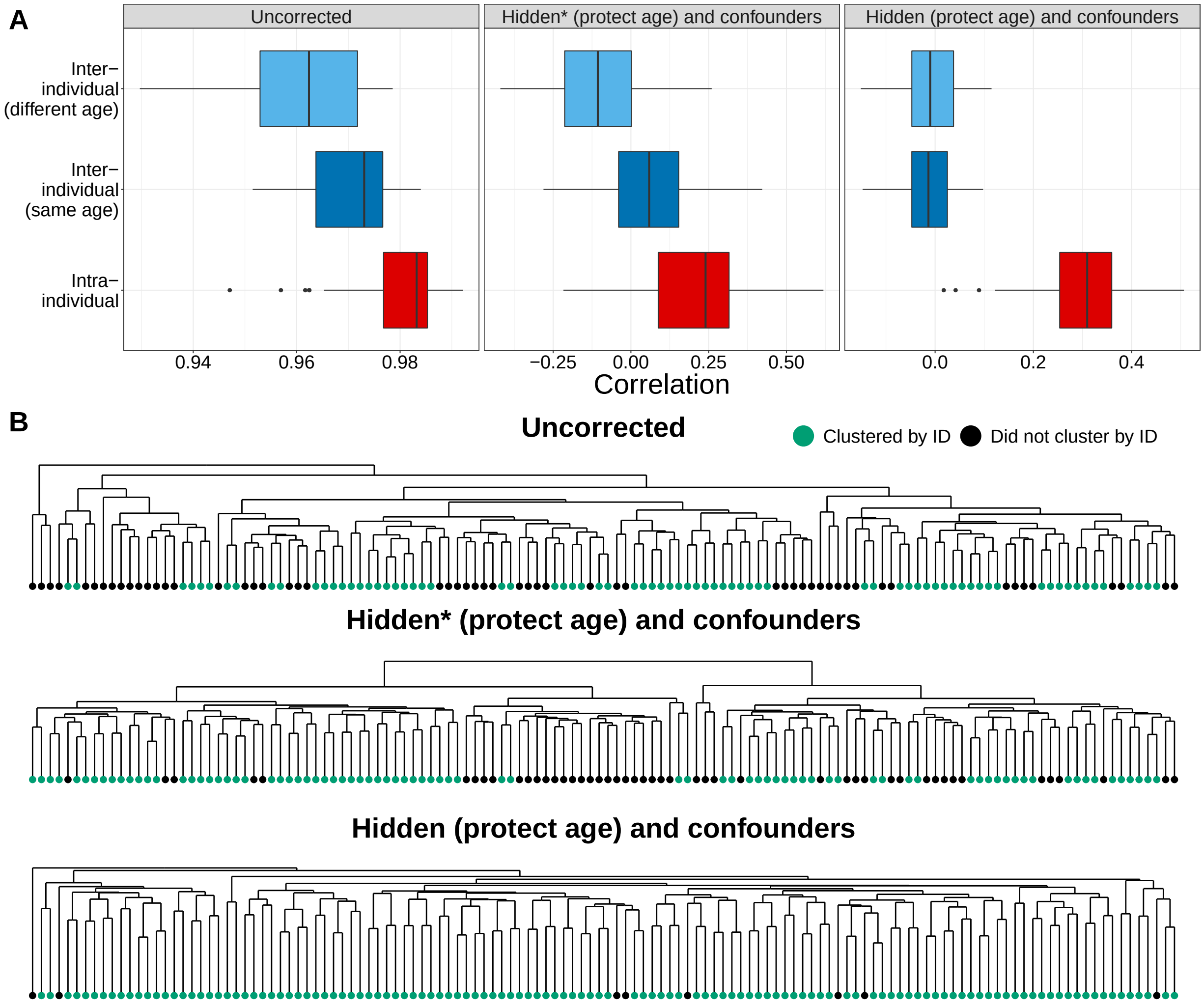

Figure S4: **Gene expression within individuals is highly correlated.** **(A)** Intra- and inter-individual gene expression correlations (Spearman's ρ; across all genes) based on uncorrected data (Uncorrected), data corrected for confounders and all inferred components of gene expression variability (Hidden (protect age) and confounders) or confounder and only inferred components that are uncorrelated with age (Hidden* (protect age) and confounders). Intra-individual (red) refers to samples of the same individual at the two ages. Inter-individual refers to samples of two different individuals that share age (dark blue) or do not share age (light blue), for 65 randomly sampled pairs of individuals. Even after correcting for global determinants of gene expression variability, the intra-individual correlations are higher than the inter-individual correlations, due to cis genetic and environmental effects that are unique to the individuals. **(B)** Dendrogram of expression-based sample-to-sample distance. Measurements of the same individual at the two ages cluster together (green) for the majority of samples. Labels of nodes denote sample ids; odd number ids are age 70 samples while even are age 80. Pairs of consecutive ids refer to the same individual, e.g. 3 and 4 refer to the same individual at age 70 and 80.

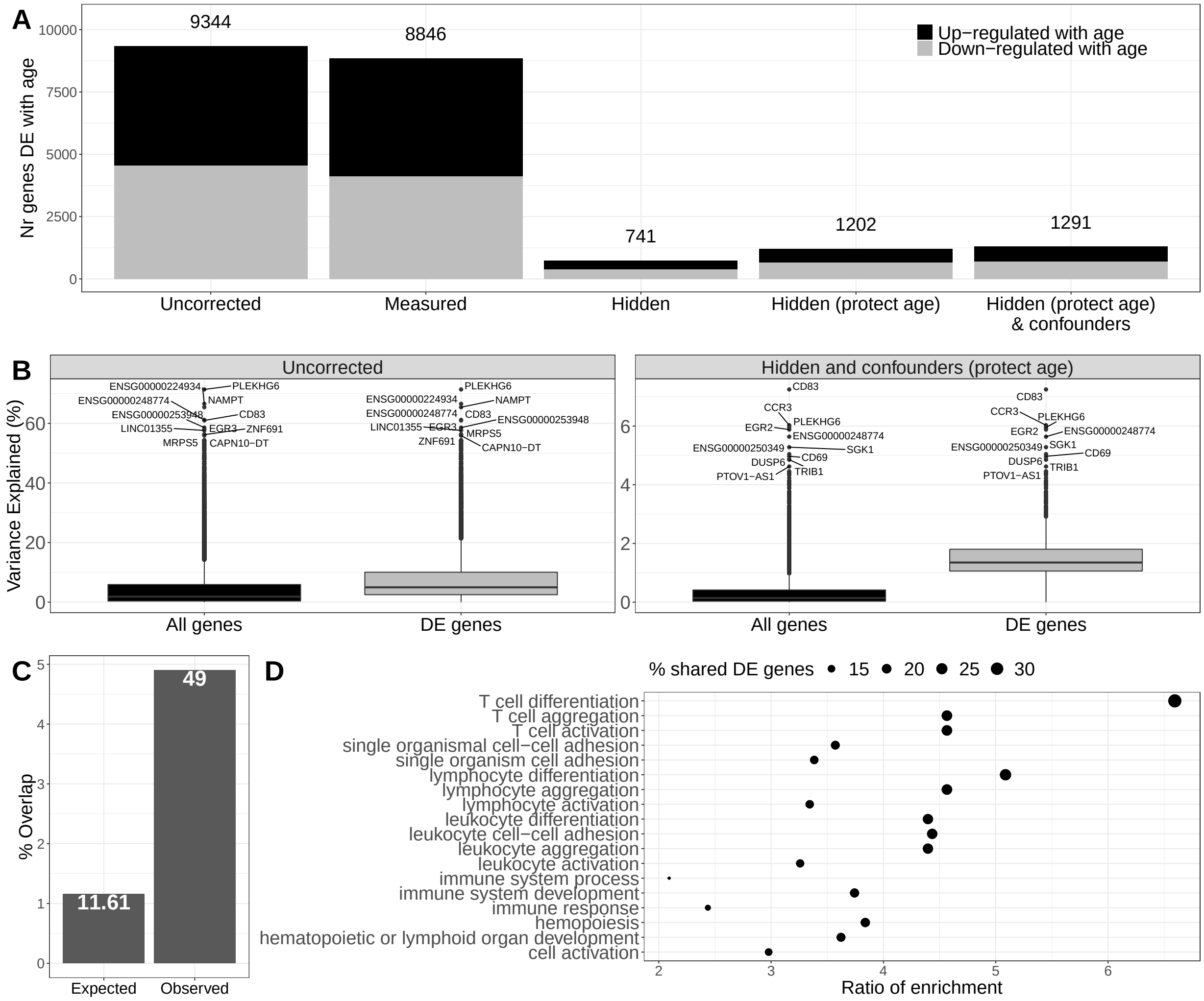

Figure S5: **Population-level age-specific expression across the transcriptome.** **(A)** Proportion of tested genes differentially expressed (DE) with age and DE genes that show down-regulation with age (FDR $\leq$ 5%) for different background noise correction methods. Measured factors are listed in Table S1. Confounders are RIN and RNA-concentration. Numbers on top of bars show number of DE genes. **(B)** Box-plot of expression variance explained by age (in %) across all genes (black) or significantly DE genes (grey). In uncorrected data, age explained, on average, 4.8% of the expression variance of all genes (median=1.84%) and 7.9% (median=4.97%) for genes DE with age. In the data corrected for hidden factors and confounders, age explained a smaller proportion of expression variance (note the difference in y-axis scale), since we removed part of expression variance attributed to age that could be due to confounders. Globally, age explained, on average, .31% (median=.14%) of expression variance, while for genes DE with age, it explained 1.5% (median=1.3%) of expression variability. **(C)** Proportion of overlap between the top 1,000 DE genes in PIVUS with the top 1,000 DE genes from CHARGE and SardiNIA. We found a statistically significant overlap between PIVUS and the other two studies (Fold enrichment = 4.17; Hyper-geometric exact test; $P = 1.3 \times 10^{-16}$). **(D)** Gene ontology enrichment analysis for the 49 genes that are in the top 1000 most DE genes across all three studies.

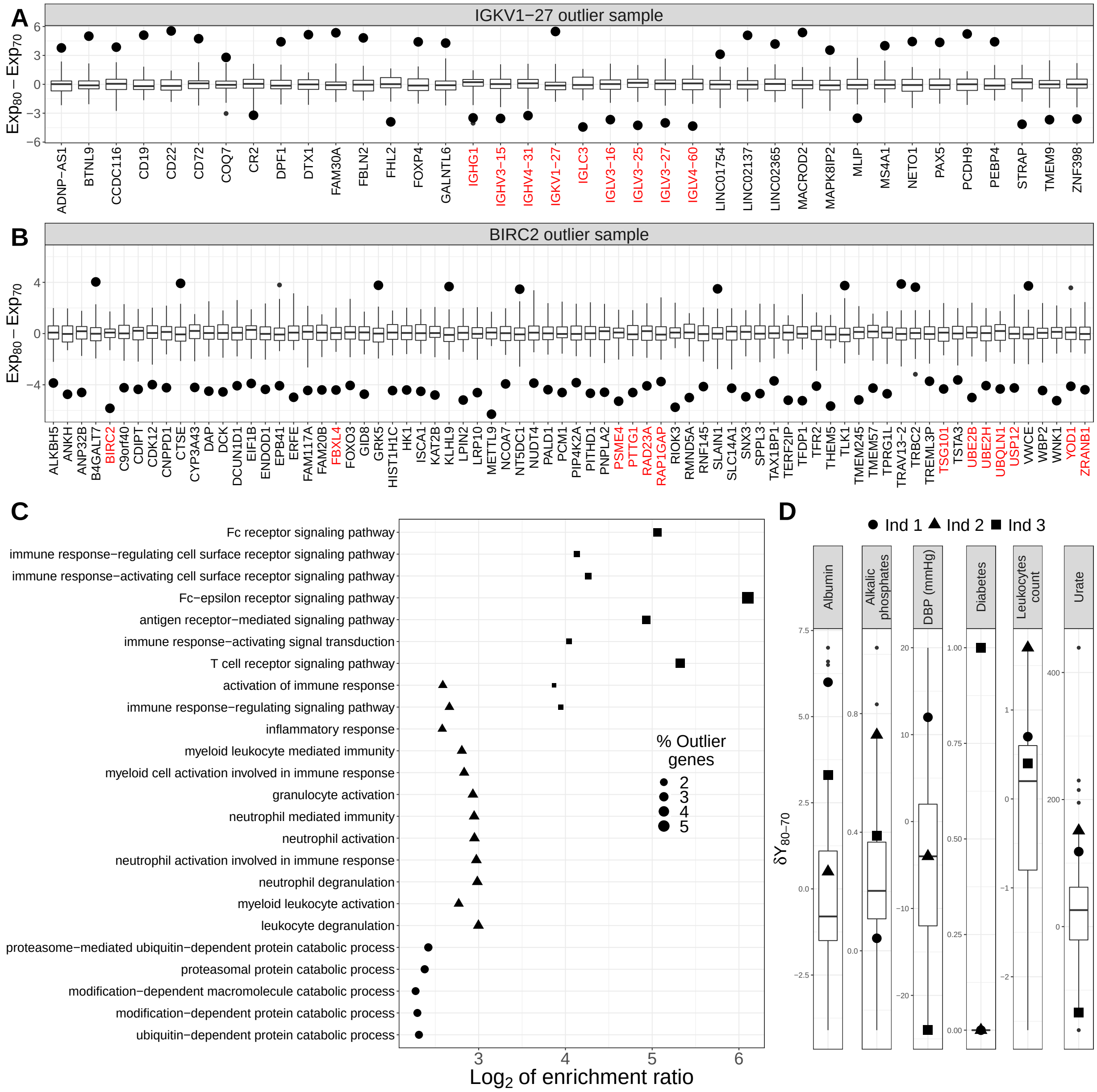

Figure S6: **Examples of age-trajectory outliers.** **(A)** Outlier genes for the individual that showed the largest outlying expression increase with age in *IGKV1-27*. The same individual was an outlier for several additional immunoglobulin-related genes (in red). Y-axis shows the expression differences between two ages. **(B)** Outlier genes for the individual that showed the largest outlying expression decrease with age in *BIRC2*. The same individual was an outlier for several other genes related to proteasomal protein catabolic process (in red). **(C-E)** Enrichment for GO biological processes for each individual's set of genes with outlying decrease of expression with age (C) and outlier phenotypes for the individuals with significant enrichment of GO terms (E). We observed significant enrichment (FDR $\leq 5\%$) for known age-related GO terms for three individuals. For two of these individuals, one of which showed a large increase in leukocyte counts between the two ages, we see enrichment for terms related to immune response[24]. For the third individual, we see enrichment for terms related to protein catabolism[25]. The same individual had a substantial increase in albumin levels between the two ages, and was diagnosed with diabetes between age 70 and 80. Y-axis in D shows the phenotype differences between two ages (δY_{80-70}). The larger symbols indicate where the outlier individual is located in the distribution of each gene/phenotype.

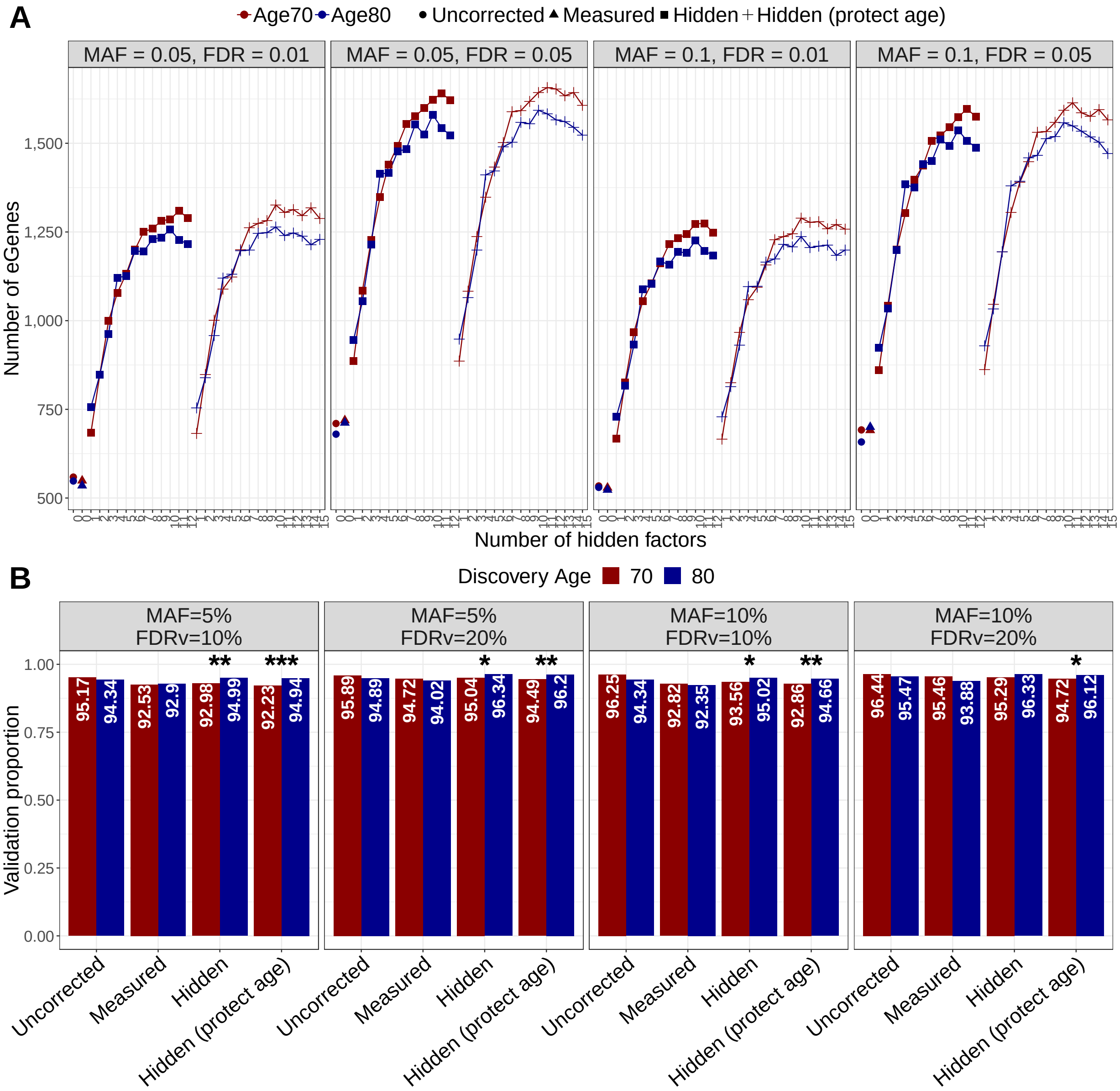

Figure S7: **Age-specific genetic regulation across the genome.** **(A)** Number of genes with at least one significant eQTL (eGenes) at age 70 (red) and 80 (blue) for different background noise correction strategies (shape), minor allele frequencies (MAF), and FDR thresholds used to identify eGenes and eQTLs[26]. We find the largest number of discoveries when we correct for hidden factors. For all MAFs and FDR thresholds we make more discoveries at age 70, compared to age 80. **(B)** Proportion of eGenes discovered at age 70 (80) that validated at age 80 (70) for different background noise correction strategies, validation FDR levels (FDRv), and MAF filters for candidate eQTL at each age. The discovery FDR was 1%. *, **, and *** indicate that the validation proportion at age 70 is lower than the one at age 80 at $\leq$10%, 5%, and 1% nominal significance levels, respectively.

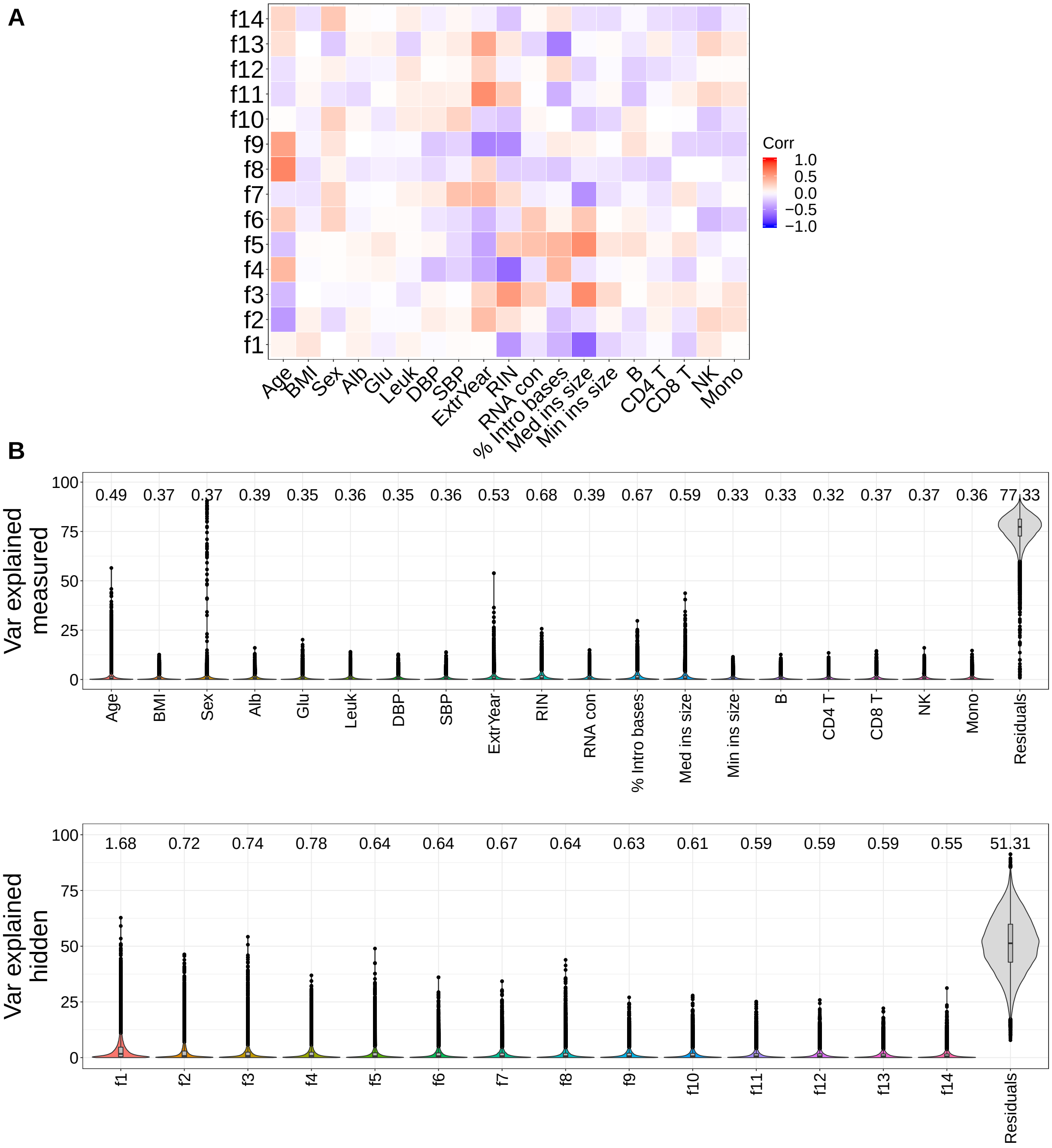

Figure S8: **Known and inferred determinants of alternative splicing variability**. **(A)** Correlation between measured covariates and alternative splicing hidden factors. Hidden factors are correlated with several measured factors. Several hidden factors are correlated with age, e.g. Spearman's $\rho_{f_8,age} = 0.53$. **(B)** Proportion of alternative splicing variance explained (VE) by measured and hidden factors. VE for each measured or hidden factor is estimated by fitting a multiple linear mixed model with all measured or hidden factors for each gene. VE by the measured or hidden factors for alternative splicing is lower than the VE for expression. This is due to intron excision ratios being internally normalized and thus less affected by technical variability.
