# Supplementary material for: Genetic regulation of gene expression and splicing during a 10-year period of human aging
Source: Genome Biol. 2019 Nov 4;20:230. doi: 10.1186/s13059-019-1840-y (PMC6827221; doi:10.1186/s13059-019-1840-y)
Supplement: Supplementary file 12 — Additional file 12 Review history. [file 13059_2019_1840_MOESM12_ESM.docx]

Review History

**First round of review**

**Reviewer 1**

**Are you able to assess all statistics in the manuscript, including the appropriateness of statistical tests used?**

Yes, and I have assessed the statistics in my report.

**Comments to author:**

Balliu et al. studied changes in gene expression, splicing patterns and allele-specific expression late in life using the PIVUS cohort. In general, the manuscript is well written and easy to follow.

The originality of the study is that the experimental design is a longitudinal study, whereas most, but not all, similar former studies were cross-sectional.

The data indicates that the genetic control of gene expression and splicing reduces slightly with age, likely due to increase influence of environmental effects and dysregulation of the DNA repair pathways. This is the major claim of the study. This point makes intuitive sense and probably indeed holds true. However, some moderation of this claim should be made due to the experimental design. Also, this finding should be discussed against previous longitudinal studies.

There is little novel insight regarding ageing biology. Nonetheless, the expression and splicing analysis results could constitute an interesting resource for ageing specialists.

The authors also report analysis of age and individual-specific expression and splicing (outliers). This part seems weak due to lack of replicates (see below).

Altogether, I recommend a major revision of the manuscript. The manuscript is interesting for the systems genetics and quantitative genetics community and probably for the Genome Biology readership.

Major points:

------

On page 2 line 32, the authors claim that there is no complete picture on longitudinal effects of aging on gene expression and splicing and their genetic regulation. However, Bryois et al. (10.1101/gr.207688.116) already showed the loss of genetic regulation with age using a longitudinal study. The sample size was lower and timespan shorter but the data was based on twins. Also, Wang et al. (10.1038/s41598-018-29086-2) did a differential splicing analysis across 10 samples with a 15 years' time difference as well as an analysis across GTEx tissues. The latter paper was not discuss at all and only mentioned in the introduction. Hence, detailed discussions of these two papers should be made to clarify the contribution of the present study.

The comparison between old and young samples may be confounded by any other covariates correlating with the two campaigns. This should be underscored.

The comparison of significant eQTL numbers could also reflect a change in statistical power. For instance the RIN seems to correlate with age. FigS1A all RIN<6 are age 70 and all RIN>8 are 80. The study does not seem to have replicates. Otherwise, it should be checked whether the between-replicates variance is larger for the later campaign (80-year old individuals) than for the earlier campaign (70-year old samples). The same caveat applies to other observations including the number of eGenes and the drop of cis-heritability. Also, the increase in allelic imbalance could reflect increase of noise. At the very least the authors should discuss such potential confounding effect.

To circumvent the potential difference in statistical power between the 70-year and the 80-year old samples, what about doing i) eQTL mapping jointly using both the 70 and the 80 old data, ii) scatter plotting the effects at 70 vs 80, and iii) computing a PCA to estimate the trend (a regression would suffer from regression-towards-the-mean effect). Is there a general reduction of effect sizes at 80 vs 70 as exemplified in 3D and 3E?

The section 2.2 "Individual-level age-specific expression across the transcriptome" is problematic. It is unclear what the impact and interpretation of outlying gene expression is in the context of ageing. This is in contrast to Li et al Nature 2017 who relate expression outliers to rare regulatory variants and Brechtmann et al AJHG 2018 who relate them to rare disease diagnostics. The analysis of outlying profiles is based on only two time points per individual without replicates. How can these outliers be related to aging and not any other difference (e.g a specific state of the individual when the blood sample is collected such as an infection, the meal the day before, temperature this day, etc.). The analysis of outliers could either be removed or investigated in more details so that one understands it biological basis. How does the number of outliers per sample distribute? Are there sample-specific pathway enrichment? The same issue applies to Individual- and age-specific ASE and splicing.

There is no significance cutoff applied to detect expression outliers.

In multiple parts of the analysis (p3 l43, p5 l35-37, p6 l60, p8 l35, …), different FDR cutoffs are used. This is confusing for the reader and also raises the question why a simple single cutoff was not sufficient. This is explained on page 5 line 48, but we would recommend to use one single FDR cutoff - eg. 5% - across the paper for the primary analysis and 20% for the validation. This would make it cleaner, especially since the authors claim in figure S7B that different cutoffs do not affect the results.

On page 4 line 15 the authors find 49 DE genes which do overlap with the CHARGE and SardiNIA dataset. But we assumed from the text that they also would have the same directionality. From the supplementary table S5 16 out of 49 genes disagree on the direction. Especially, if one is doing a GO enrichment on the overlap, the directionality should be taken into account. Also the differences between the findings should be discussed in the paragraph.

Minor points:

--------

The slightly larger proportion of differentially expressed (DE) genes showing down-regulation with age is quite dependent of the normalization procedure. Did the authors used spike-ins? I suggest to moderate this claim.

The overlap of DE genes is significant but very low (5%). The authors should comment why it is so low.

"The proportion of eGenes discovered at age 70 was significantly smaller than the proportion of eGenes discovered at age 80" . The effect size (fold change) should be given along with the significance.

Fig 1C would be better displayed in two siamese halves for the positive and negative signs. The up and down triangle shapes are hard to distinguish.

Fig 2: To gain an overview of the outlier distribution one could add a violin plot to panel B. For panel C I would suggest the same as for Figure 1C.

Fig 3B could better be displayed as a Venn diagram labeled with counts and relative proportions. With a quick glance, the current representation suggests stronger eQTL signal at 80.

Fig 3C: What statistical test was performed? Is it a paired test? The data could better be shown with a scatterplot or a boxplot of the heritability ratios.

Pi is between [0,1]. It is not the allelic ratio but "the proportion of reads supporting the reference allele". It could be called "reference allele proportion".

The moderate correlation in Fig 4A can be explained by noise as ratios are unstable especially close to 0.5 where count-based proportions have largest variance. This should be noted since the text now might suggest this is due to an age-related change.

In discussion, p.9 line 30: argument of biological age differing from the chronological age also applies to the duration. 10 years apart for one individual does not correspond to 10 years part for another individual. So the longitudinal design only mitigate this issue.

Figure S6: Please add an explanation of the red coloring of the gene names in panel A-C.

**Reviewer 2**

**Are you able to assess all statistics in the manuscript, including the appropriateness of statistical tests used?**

Yes, and I have assessed the statistics in my report.

**Comments to author:**

The manuscript by Balliu et al. reports results from one of the first long-term longitudinal studies in humans examining the role of age and genetic variation on gene expression and alternative splicing in healthy older adults. By performing RNA-seq on whole-blood from the same 65 individuals at age 70 and 80, the study identifies differentially expressed and alternatively spliced genes between the two age groups. eQTL mapping and allele-specific expression analyses are used to quantify the effects of aging on the genetic regulation of gene expression and alternative splicing. Throughout the manuscript, the authors highlight genes which are differentially regulated and are involved in biological processes that have been implicated in aging. Overall this is an interesting dataset with potential to investigate important regulatory changes during ageing with a paired study design. The differential gene expression and splicing analyses present strong evidence for the effect of age on gene regulation. This could be further expanded and strengthened by adding potential mechanistic insight with a similar analysis performed on the methylation data. However the authors use methylation exclusively to correct for changes in cell composition with age.

The analysis of changes in the genetic regulation of gene expression with age, though potentially of high interest, is not convincing (eQTL, sQTL) or identifies a limited number of changes (ASE analysis) thus limiting the overall impact of the results and the ability to identify general trends. In general the authors should be more cautious in interpreting their results and presenting their conclusions. Detailed comments are reported below.

-One potential problem in studies that compare gene expression or other variables between two groups is the presence of batch effects that may create false positives. The manuscript overall presents careful analyses in assessing background noise and potential technical variables. However, there is no information presented on the strategy utilized to prevent any batch effect due to RNA extraction and library preparation. Was the study design balanced between age groups, with respect to these technical aspects?

-Another important covariate is sex. No information is provided about the sex of the samples and sex is not included as a covariate in the model, despite using both the X and Y chromosome in the differential expression analysis. How did the authors handle this important covariate? Ageing is a biological process that presents important differences between men and women. It would be interesting to see an analysis of sex x age effects on gene expression, if enough samples were included from each sex.

-In Figure S1, it appears clear that the distribution of RIN is different between the two age groups. Indeed RIN is one of the most important variables included in the gene expression model. Despite accounting for RIN, it is possible that residual differences in RNA degradation may account for some of the DE genes observed between the two age groups. Please include some additional analyses demonstrating that DE genes are not enriched for long genes or a 3' bias.

-A subset of DE genes are reported to be associated with complex traits. What are the complex traits the genes are associated to? Even though a full list is reported in the supplementary table, it would be helpful to include a summary of the most represented traits and interpret the biological significance of these results in the context of ageing.

-Are DE genes enriched for eQTLs? Are they under stronger/weaker genetic control, compared to non-DE genes?

-Are the different statistical tests well calibrated? Please include QQ-plots for the p-values form the DE and differential splicing analyses and also for the eQTL and sQTL analyses.

-As mentioned above, it is surprising that the authors do not investigate if there are differences in methylation with age. Maybe such differences (if found) could explain some of the mechanisms underlying differential gene expression.

-For the eQTL mapping analysis, were permutations run to calibrate the p-value distribution?

-Age-specific eQTL mapping. This is the most problematic aspect of this manuscript. A formal linear model to test for age x genotype effect is needed to convincingly demonstrate that genetic regulation of gene expression changes over time. While the method employed is valid to identify eQTLs that replicate across age groups, the 20% FDR threshold is too strict to confidently determine the absence of genetic regulation on gene expression. Lack of replication doesn't necessarily imply age-specific effects. It would also help to see a plot that compares the genetic effect (beta) between ages, similar to the plot for pi in figure 4 A. A similar issue applies to the sQTL analysis.

-What is the interpretation of a difference in 1% of the heritability of gene expression across age groups?

-Please include more details on the different analyses of ASE at the population and individual level using EAGLE.

-In the ASE analysis, was the entire gene haplotype considered or each individual SNP was considered independently?

-Discussion. "Here, we report a global reduction in genetic control and a reduction in gene expression heritability with age." This is a strong statement and the results provided do not convincingly support it. Less strong language should be used.

Minor Comments:

Pg. 7 Line 54: This should be Fig. 5A instead of Fig. 5B.

This sentence is unclear: "Moreover, 18 of these DE genes are previously known to be complex-trait associated genes where gene expression levels modulate disease risk in whole blood (Sup Methods and Tab. S4)." Pg. 3 Line 50. Please re-write: Moreover, 18 of these DE genes are associated with complex-traits (reference) where gene expression levels in whole blood modulate disease risk (Sup Methods and Tab. S4).

Figure 1B, C, 2C. The triangles are not easy to distinguish. I suggest using 2 different colors but same shape for the up and down regulated genes. Or 2 different shapes that are easier to distinguish.

In figure 4D, one individual goes in opposite direction for the first 2 SNPs. What's the interpretation?

Please check that supplementary tables and figures are numbered in the order they are referenced in the text.

**Authors Response**

**Point-by-point responses to the reviewers’ comments:**

We thank the reviewers and editor for the very helpful comments. Please find a detailed response below.

Reviewer #1:

**Major** **points**:

1. On page 2 line 32, the authors claim that there is no complete picture on longitudinal effects of aging on gene expression and splicing and their genetic regulation. However, Bryois et al. (10.1101/gr.207688.116) already showed the loss of genetic regulation with age using a longitudinal study. The sample size was lower and timespan shorter, but the data was based on twins. Also, Wang et al. (10.1038/s41598-018-29086-2) did a differential splicing analysis across 10 samples with a 15 years' time difference as well as an analysis across GTEx tissues. The latter paper was not discussed at all and only mentioned in the introduction. Hence, detailed discussions of these two papers should be made to clarify the contribution of the present study.

Thank you for your comment, we now discuss the previous literature on longitudinal studies of aging in more detail in the introduction on page 2, lines 9-16 (new text in bold below).

“The impact of aging on gene expression and genetic regulation has been well-studied in model systems, such as yeast, fruit fly or worm ^4-5^ while much less is known about the transient nature of gene regulation and expression in humans. The majority of studies that have been performed in humans have been cross-sectional ^6-15^. The few longitudinal studies have either focused on a specific disease or intervention ^16-21^, looked over a short time span ^22^, or focused on cell lines ^23^. **For example, *Bryois et al***^22^ ***studied differences in RNA sequencing-based gene expression levels in whole-blood from female twins of the UK Adult Twin Registry longitudinally at two time points separated, on average, by 22 months. At each time point, the individuals varied in age from 45 to 80 years old.*** ***Harries et al*** ^23^ ***studied the impact of aging on array-based gene expression levels in lymphoblastoid cell lines derived from members of the Lothian Birth Cohort 1936 at mean ages 70 and 76 years. The impact of aging on genetic regulation of gene expression was not examined in this study.*** Even less is known about the effect of age on alternative splicing and its genetics, even though changes in alternative splicing have previously been linked to aging-associated phenotypes^14,24-27^. As a result, a complete picture of the long-term effect of aging on gene expression and splicing and their genetic regulation in humans is still lacking**, especially late in life.**”

We also mention the Harries et al 2017 results in the section about differential expression on page 4, lines 62-63 (new text in bold below).

“We investigated transcriptome-wide changes in gene expression with age (Fig. 1B and S5A and Tab. S2). We discovered 1,291 genes (8% of tested genes) whose expression levels were significantly associated with age (FDR ≤ 5%). **Harris et al observed a similar number of differentially expressed genes between ages 70 and 76 years in lymphoblastoid cell lines.** The DE genes showed significant enrichment (FDR ≤ 5%) for multiple aging-related pathways (Fig. 1C and Tab. S3) ”

We do not discuss the Wang et al. study in the context of longitudinal studies since they performed the differential splicing analysis in GTEx (cross-sectional study). The 10 longitudinal (male) samples were only used to test their splicing-based age predictor.

2. The comparison between old and young samples may be confounded by any other covariates correlating with the two campaigns. This should be underscored.

The reviewer raises a really important comment about longitudinal studies. Due to the design of such studies, comparisons between the two age groups could also reflect the effect of confounders, i.e. variables correlated with both age and gene expression. We took the following steps in order to minimize the effect of confounders: First, we minimized confounding effects by study design, i.e. blood samples from each age were frozen immediately upon collection (in 2001-2004 for age 70 samples and 2011-2014 for age 80 samples). The RNA extraction, library preparation, and sequencing for the same individual was always performed in pairs and by the same technician. Second, we identified and corrected our analyses for measured biological or technical variables that could act as confounders by checking if they are correlated with age and gene expression, in our case RNA Integrity Number (RIN) and RNA concentration. Third, we inferred and corrected for unmeasured/hidden factors of expression variability, allowing such factors to be correlated with both expression and age. For example, we corrected for hidden factors 4-5 which were moderately correlated with age (Figure S3A) and could be capturing unmeasured confounder effects. This is a conservative strategy since such factors could also be capturing part of the aging effect, but we believe it is necessary to avoid confounding effects by unmeasured factors (e.g. impact of freezing time). This strategy explains also the large difference in the number of differentially expressed genes between the uncorrected analysis or analysis corrected only for measured factors and our final analysis which corrects for measured and unmeasured potential confounders (Figure S5A). To underscore the impact of background noise / confounders in our analyses and longitudinal studies in general, we have added the following sentences to the results, methods, and discussion sections of our manuscript (new text in bold):

Results section - Page 3, lines 43-49: “***Background noise correction, the process of identifying and correcting for major components of expression variability, unrelated to the variable of interest, e.g. RNA library size or cell-type composition, is crucial in (longitudinal) RNA-Seq experiments in order to improve power and avoid false discoveries. We performed extensive background noise correction for each analysis (see Methods and Supplemental Methods). Unless otherwise mentioned, all results are based on analyses corrected for measured and inferred components of gene expression variability.*** ”

Results section - Page 5, lines 90: “Age explained 1.5% of expression variance for genes significantly associated with age. This estimate is smaller than the estimate from the uncorrected analysis of our data (average across DE genes = 7.9%), **due to our conservative background noise correction approach,** but comparable to estimates from other aging studies in humans, e.g. 2.2% in Viñuela et al.^14^.

Methods section - Page 14, lines 384-400: “**It is well known that major components of expression variability can often be attributed to technical factors, e.g. RNA library size, that introduce unwanted, systematic variability in the data. Recovering and correcting for such factors can improve power to find differentially expressed/spliced genes or identify e/sQTLs. When these factors are correlated to the variable of interest, in our case age, they can act as confounders and correcting for such factors is necessary in order to control bias and prevent false discoveries.**

**To identify potential measured confounders, we search for measured factors that are associated with both age and gene expression. RIN and RNA concentration are moderately correlated with age (Spearman's ρ = -0.46 and -0.30, Figure S3) and they are both associated with gene expression (π_1_ = 61.43% and 22.19%, Table S1).** To extract and correct for **unmeasured** components of gene expression variability **(referred to as *hidden factors*)**, we used surrogate variable analysis (SVA) as implemented in the R package *smartSVA*^67^. We set age as variable of interest **and allowed for the factors to be correlated to age** (Fig. S3). **While the latter could reduce our power to identify differentially expressed genes, when correcting our analysis for these factors, it is a necessary step to reduce false positive associations.** More details about the implementation of SVA can be found in the Supplemental Methods. We selected SVA, as opposed to other background noise correction methods^68-69^, on the basis of recent work that shows it is robust to spurious associations when setting a variable of interest^70^.”

3. The comparison of significant eQTL numbers could also reflect a change in statistical power. For instance, the RIN seems to correlate with age. Fig S1A all RIN<6 are age 70 and all RIN>8 are 80. The study does not seem to have replicates. Otherwise, it should be checked whether the between-replicates variance is larger for the later campaign (80-year old individuals) than for the earlier campaign (70-year old samples). The same caveat applies to other observations including the number of eGenes and the drop of cis-heritability. Also, the increase in allelic imbalance could reflect increase of noise. At the very least the authors should discuss such potential confounding effect. To circumvent the potential difference in statistical power between the 70-year and the 80-year old samples, what about doing i) eQTL mapping jointly using both the 70 and the 80 old data, ii) scatter plotting the effects at 70 vs 80, and iii) computing a PCA to estimate the trend (a regression would suffer from regression-towards-the-mean effect). Is there a general reduction of effect sizes at 80 vs 70 as exemplified in 3D and 3E?

To minimize the impact of differences in statistical power between the two ages, we find and correct for the number of unmeasured hidden factors that maximizes eQTL discovery *separately at each age*. In the case of RIN, one of the hidden factors we corrected the analysis for is correlated to RIN and should hence remove any differences in power related to RIN. In addition, to minimize false negative discoveries, we use a strict FDR threshold for eQTL discovery, but several lenient FDR thresholds for eQTL validation. The between-replicates variance is higher at age 80, compared to age 70, before correcting for background noise (“Raw data” in the plot below) but the difference is much smaller after we correct for hidden factors (final data in which we call eQTLs; “Residuals” in the plot below).

Furthermore, to address potential differences in statistical power, we show that eQTL discovery for both ages is different across a range of hidden factor corrections for each (Fig 3A). We think this analysis effectively shows that we are robust to differences in statistical power as correcting for *any* hidden factor number from 7-15, eQTLs discovered at 80 are lower than discoveries made using *any of* hidden factors 7-15 for age 70 eQTL discovery.

Joint eQTL mapping at both ages would require the use of a mixed model in order to take into account within-individual correlation due to shared genetics and environment. Unfortunately, mixed models are computationally intensive and are hard to scale for eQTL mapping. Ignoring such correlations (by running a simple linear model across ages) could lead to decreased standard errors of the eQTL effect sizes and thus increased false positive rates. As an alternative, we compared the effect sizes at each age for the genes with eQTLs at both ages and the genes that showed loss of genetic regulation with age. The plot below shows the eQTL effect sizes for all genes with eQTLs at both ages (in black) and the effect sizes for the genes with age eQTLs only at age 70 (in dark red). The dotted line indicates the identity line (x=y). The solid black and black lines indicate the regression line for genes with eQTLs at both ages and genes with eQTLs only at age 70.

For genes with eQTLs at both ages, the eQTL effect sizes at age 70 are significantly larger, on the absolute scale, than the eQTL effect sizes at age 80 (Wilcoxon-signed rank test; (pseudo-)median(|beta80|-|beta70|)= -0.0043, p-value < 2.2e-16, calculated across 209,677 significant gene - SNP pairs). Moreover, for genes with eQTLs only at age 70, the effect sizes at age 70 are significantly larger than the effect sizes at age 80 (Wilcoxon-signed rank test; (pseudo-)median(|beta80|-|beta70|)= -0.25, p-value < 2.2e-16; calculated across 6,015 significant gene - SNP pairs at age 70), indicating that our observation is unlikely to be only due to increased technical noise. We now include this plot as part of the main figure presenting the eQTL results (Figure 3C).

We have made the following modifications to the manuscript to discuss the impact of differences in statistical power between the two ages (new text in bold).

Results section - Page 7, lines 151-153: “We evaluated the association between gene expression at each age group and genetic variants within 1Mb of the transcription start site using linear regression (Methods). **To minimize the impact of differences in statistical power between the two ages, we find and correct for the number of hidden factors that maximizes eQTL discovery separately at each age (Fig. 3A and S7).** After background noise correction, we detected significant eQTLs for 1,326 genes at age 70 (8.5% of tested genes, FDR≤1%, Tab. S8), 92.2% of which replicated at age 80 (FDR≤10%, Fig. 3B). **The stringent FDR cutoff for discovery ensures low proportion of false positive eGenes, i.e. genes with at least one significant eQTL, at each age and the liberal FDR for validation ensures low proportion of false age-specific eGenes.** “

Results section - Page 7, lines 165-172: “**We compared the eQTL effect sizes at each age for genes that are eGenes at both ages (shared) and genes that are eGenes only at age 70. For shared eGenes, the difference in eQTL effect sizes between the two ages is small but significant (Wilcoxon-signed rank test; Median(|β80|-|β70|)= -0.0043, p-value < 2.2e-16) due to the large number of significant eQTLs from which the difference is calculated (approximately 210K). In addition, for genes that are eGenes only at age 70, the effect sizes at age 70 are significantly larger than the effect sizes at age 80 (Wilcoxon-signed rank test; Median(|β80|-|β70|)= -0.25, p-value < 2.2e-16).** To compare the effect sizes genome-wide, we estimated the *cis* heritability for each gene ... ”

Figure 3C and its legend: “**Scatter plot of eQTL effect sizes at each age for genes that are eGenes at both ages (shared) and genes that are eGenes only at age 70. For shared eGenes, the difference in eQTL effect sizes between the two ages is small (Wilcoxon-signed rank test; Median(|β80|-|β70|)= -0.0043, p-value < 2.2e-16). On the other hand, for age 70 eGenes, the effect sizes at age 70 are significantly larger than the effect sizes at age 80 (Wilcoxon-signed rank test; Median(|β80|-|β70|)= -0.25, p-value < 2.2e-16).**”

4. The section 2.2 "Individual-level age-specific expression across the transcriptome" is problematic. It is unclear what the impact and interpretation of outlying gene expression is in the context of ageing. This is in contrast to Li et al Nature 2017 who relate expression outliers to rare regulatory variants and Brechtmann et al AJHG 2018 who relate them to rare disease diagnostics. The analysis of outlying profiles is based on only two time points per individual without replicates. How can these outliers be related to aging and not any other difference (e.g. a specific state of the individual when the blood sample is collected such as an infection, the meal the day before, temperature this day, etc.)? The analysis of outliers could either be removed or investigated in more details so that one understands it biological basis. How does the number of outliers per sample distribute? Is there sample-specific pathway enrichment? The same issue applies to individual- and age-specific ASE and splicing.

We agree with the reviewers perspective that it is hard to understand the basis of such trajectories. Our age-trajectory outlier analysis was meant to capture differences in the rate of aging among individuals and to compliment the DE analysis which is based on chronological rather than biological age. The existence of age-trajectory outliers is not necessarily in conflict with the rare variant hypothesis; age-trajectory outliers could be a result of rare variants (in environmentally-responsive TSS) that interact with aging to alter gene expression. Unfortunately, the absence of DNA sequencing data does not allow us to investigate this hypothesis and further, we did not expect that rare variants would be the major source of trajectory differences.

As the reviewer points out, due to our study design, it is challenging to show that the outliers are (only) due to aging and not due to other individual-specific differences between the two time points. In an attempt to understand the biological basis of personal expression outlier genes, we now include the distribution of the number of outliers per individuals in the main figure (Figure 2C; also shown below). In summary, the median number of outlier genes per individual was 4 with 14% of individuals having no outliers. The maximum number of outlier genes for an individual was 76. In addition, we performed Gene Ontology (GO) enrichment analysis for the outlier genes of each individual separately (Figure 2C and S6C; also shown below), as well as contrast them with outlying covariate / phenotype data for the individual (see Figure S6D; also shown below). We observed significant enrichment (FDR<5%) for specific GO biological terms for the outlier genes of three individuals. As expected, these are the individuals with a large number of outlier genes. For two of these individuals, we see enrichment for GO terms related to immune response. For the third individual, we see enrichment for terms related to protein catabolism. We have now moved this Figure to the main manuscript (Figure 2D) in order to give some insight regarding the biological basis of these outliers.

Figure 2:

Figure S6:

We have updated the Results section as follows:

page **5-6**, lines **118**-**121** (new text in bold): “The longitudinal design of our study enabled us to also investigate changes in individual-level expression profiles with age. **While such changes compared to population-level changes can be due to any number of personal environments (i.e. disease, diet, medication), we hypothesized that outlier trajectories may reflect some individual-specific differences in aging.** We searched for individuals with outlying age trajectories … ”

page **6**, lines **127**-**128** (new text in bold): “We searched for individuals with outlying age trajectories (schematically illustrated in Fig. 2A) and found 555 individual-gene outlier pairs from 529 unique genes (Fig. 2B and Tab. S7); … **The median number of outlier genes per individual was 4 with 14% of individuals showing no outliers while 13%**

**had more than 20 outlier genes (Fig. 2C)**.”

page **6**, lines **136**-**145** (new text in bold): “**To aid the understanding of the biological basis of personal expression outlier genes**, we performed enrichment analysis of GO biological processes **for the outlier genes of each individual** **(Fig. 2D and S6C),** **as well as contrast them with outlying covariate / phenotype data for the individual (Fig. S6D)**. For genes with outlying decrease of expression with age, **we observed significant enrichment (FDR<5%) for known age-related GO terms for three individuals. For two of these individuals, one of which showed a large increase in leukocyte counts between the two ages (Fig. S6D), we see enrichment for terms related to immune response. For the third individual, we see enrichment for terms related to protein catabolism. The same individual had a substantial increase in albumin levels between the two ages, and was diagnosed with diabetes between age 70 and 80 (Fig. S6D).** In contrast, genes in which individuals showed an outlying increase of expression with age were not enriched for any specific functions.

Figure 2 C & D legends: **“(C) Number of outlier genes for each individual. The median number of outlier genes per individual was 4 with 14% of individuals showing no outliers while 13% had more than 20 outlier genes. (D) Enrichment for Gene Ontology biological processes for outlier genes of each individual. We observed significant enrichment (FDR < 5%) for specific terms for the outlier genes of three individuals. For two of these individuals, we see enrichment for terms related to immune response. For the third individual, we see enrichment for terms related to protein catabolism.”**

Figure S6 C & D legends: “**(C-E) Enrichment for GO biological processes for each individual's set of genes with outlying decrease of expression with age (C) and outlier phenotypes for the individuals with significant enrichment of GO terms (E). We observed significant enrichment (FDR <5%) for known age-related GO terms for three individuals. For two of these individuals, one of which showed a large increase in leukocyte counts between the two ages, we see enrichment for terms related to immune response. For the third individual, we see enrichment for terms related to protein catabolism. The same individual had a substantial increase in albumin levels between the two ages, and was diagnosed with diabetes between age 70 and 80. Y-axis in D shows the phenotype differences between two ages.** The larger symbols indicate where the outlier individual is located in the distribution of each gene/phenotype.”

Regarding the personal differential ASE effects, the small number of such effects did not allow us to perform any enrichment analyses. We did not do any splicing-based outlier analyses due to the compositional nature of splicing data.

5. There is no significance cutoff applied to detect expression outliers.

The detection of expression outliers is based on the *standard definition of outlier* and the assumption of normal distribution (an assumption that we make for all the expression-based analyses by using linear regression models for our analyses), i.e. individuals are outliers for a gene, if their change in the expression of the gene between the two ages (E80-E70) falls outside the (Q1 - 3 x IQR, Q3 + 3 x IQR) range, where Q1 and Q3 are the 25th and 75th percentiles and IQR is the interquartile range of the distribution. We also run a formal outlier test using the Grubbs’ test for the presence of a single outlier and found the results to largely overlap the results from the standard definition of outliers . Of the 15,518 tested genes, we identify outliers in 286 genes (at FDR<5%; using the BH correction). Ninety-seven percent of the outlier genes identified using the standard outlier definition have nominal Grubbs’ test p-values <.05; 43% of them are significant after multiple testing adjustment, i.e. BH-adjusted p-value <0.05.

The figure below plots the number of outliers for each individual using the standard outlier definition and using the Grubbs’ test and controlling the FDR at 5%. As expected, using Grubbs’ test and limiting to genes with BH-adjusted p-value <0.05 results in less outlier genes for each individual, but the ordering of the individuals remains largely the same.

In addition, the top outlier genes using the -log10(Grubbs’s P-value) ranking (plot below) are largely similar to the top outlier genes using the standard outlier definition (Figure 2B).

Since the results and interpretation remained essentially the same using this alternative approach, we have not made any modifications to the manuscript to avoid making the manuscript too complicated. However, if the Editor and Reviewers believe that the presentation would benefit from it, we are happy to add these plots to the supplement.

6. In multiple parts of the analysis (p3 l43, p5 l35-37, p6 l60, p8 l35, …), different FDR cutoffs are used. This is confusing for the reader and also raises the question why a simple single cutoff was not sufficient. This is explained on page 5 line 48, but we would recommend using one single FDR cutoff - eg. 5% - across the paper for the primary analysis and 20% for the validation. This would make it cleaner, especially since the authors claim in figure S7B that different cutoffs do not affect the results.

We thank the reviewer for pointing this out. As mentioned by the reviewer and in the manuscript, the stringent FDR cutoff for discovery ensures low proportion of false positive eGenes at each age and the liberal FDR for validation ensures low proportion of false age-specific eGenes. To avoid confusion, we have moved that the sentence explaining the rationale of different FDR thresholds next to the introduction of the new FDR cutoff (text that was moved shown in bold below)

page 5, line 111: “After background noise correction, we detected 1,326 eGenes, i.e. genes with at least one significant eQTL, at age 70 (8.5% of tested genes, FDR≤1%, Tab. S8), 92.2% of which replicated at age 80 (FDR≤10%, Fig. 3B). **The stringent FDR cutoff for discovery ensures low proportion of false positive eGenes at each age and the liberal FDR for validation ensures low proportion of false age-specific eGenes.** On the other hand, we detected eQTLs for 1,264 genes in the 80-year-old samples, 94.9% of which replicated at age 70. The depletion of genes with at least one significant eQTL at age 80, relative to age 70, was statistically significant ...”

7. On page 4 line 15 the authors find 49 DE genes which do overlap with the CHARGE and SardiNIA dataset. But we assumed from the text that they also would have the same directionality. From the supplementary table S5, 16 out of 49 genes disagree on the direction. Especially, if one is doing a GO enrichment on the overlap, the directionality should be taken into account. Also, the differences between the findings should be discussed in the paragraph.

The reviewer raises a good point. The difference between the findings of the three studies could reflect differences in age distribution and non-linear age effects on expression; CHARGE has samples between 20 and 90 years of age, while PIVUS focuses only at ages 70 and 80. Differences could also be a result of expression assay; CHARGE is array-based while SardiNIA and PIVUS are based on sequence data, or background noise correction method; CHARGE is a meta-analyses of several European cohorts and the DE analyses was adjusted only for the few measured confounders, e.g. sex, smoking status, plate origin, RNA quality, and cell counts. Similar results, i.e. an overlap in the number and effect size directionality of DE genes, were observed between the CHARGE study and other aging studies, e.g. the Bryois et al and Harris et al studies. We now include results about the directionality of the effect sizes and discuss the difference between the findings in the Results section (text in bold below):

Page 5 - lines 101-107: “The 49 DE genes that **overlap in the top 1,000 discoveries** between the three studies (Tab. S5) are enriched in gene ontology (GO) terms related to adaptive immune response pathways that have previously been implicated in aging^34^, e.g. leukocyte cell-to-cell adhesion and terms reflecting the underlying age-related T cell biology (Fig. S5D). **Only 65% of these 49 genes change in the same direction with age across all studies. This discrepancy could be explained by the difference in the age distribution of the samples in the three studies and could reflect non-linear effects of age on expression. It could also be a result of differences in expression assay - CHARGE is array-based, while PIVUS is sequencing-based; or background noise correction method - DE analyses in CHARGE and SardiNIA were only adjusted for measured confounders, while our analyses in PIVUS also adjusted for hidden factors.**”

**Minor points**

1. The slightly larger proportion of differentially expressed (DE) genes showing down-regulation with age is quite dependent of the normalization procedure. Did the authors used spike-ins? I suggest moderating this claim.

We have now removed this sentence from page 3.

2. The overlap of DE genes is significant but very low (5%). The authors should comment on why it is so low.

The 5% overlap refers to the overlap of the top 1,000 DE genes between all three studies. Our actual estimated overlap with CHARGE and SardiNIA are 76% and 51% (π1 statistic). We have now included this information in the results (new text in bold).

Page 5 - lines 94-95: “We validated our DE genes *in silico* using summary statistics from two large cross-sectional studies of aging in human PBMCs (CHARGE) and whole blood (SardiNIA). **We estimated that 76% and 51% of our DE genes were differentially expressed with age in the CHARGE and SardiNIA cohorts, respectively. In addition,** we found a significant overlap between our top 1,000 DE genes and the top 1,000 DE genes from these other two studies …”

3. "The proportion of eGenes discovered at age 70 was significantly smaller than the proportion of eGenes discovered at age 80". The effect size (fold change) should be given along with the significance.

We now include difference in the two proportions (test statistic) together with the p-value. We have updated the text as follows on page 7 - lines 160 -165:

“The depletion of genes with at least one significant eQTL at age 80, relative to age 70, was statistically significant (Exact McNemar’s test; **OR=0.81**, p-value = 5.8×10−3). Moreover, the proportion of eGenes discovered at age 70 that replicated at age 80 was significantly smaller than the proportion of eGenes discovered at age 80 that replicated at age 70 (Binomial proportion test; **π70^{val}-π80^{val}= -2.7%**, p-value = 3.3×10−3)”.

4. Fig 1C would be better displayed in two Siamese halves for the positive and negative signs. The up and down triangle shapes are hard to distinguish.

We thank the reviewer for pointing this out, we have changed the shapes for up and down-regulated pathways in Figure 1C.

5. Fig 2: To gain an overview of the outlier distribution one could add a violin plot to panel B.

We thank the reviewer for this suggestion. We have now added a box plot showing the number of outliers per sample in Figure 2C.

6. Fig 2. For panel C I would suggest the same as for Figure 1C.

We thank the reviewer for this suggestion. We have used only one shape for Figure 2A since the directionality can be seen from the sign of the y-axis. Moreover, we have changed the shapes for Figure 2C as in Figure 1C.

7. Fig 3B could better be displayed as a Venn diagram labeled with counts and relative proportions. With a quick glance, the current representation suggests stronger eQTL signal at 80.

We thank the reviewer for this suggestion. We now display the difference in counts and relative proportions as two Venn diagrams. The new Figure 3B is shown to the right. The top Venn diagram shows the number of eGenes discovered at age 70 (outer circle) and the % that validated at age 80 (inner circle). The second Venn diagram bottom the number of eGenes discovered at age 80 (outer circle) and the % that validated at age 70 (inner circle).

8. Fig 3C: What statistical test was performed? Is it a paired test? The data could better be shown with a scatterplot or a boxplot of the heritability ratios.

We used beta regression to model the effect of age on heritability, correcting for the standard errors in the estimates of heritability. We tested for a difference in heritability with age using a Wald test (described in section 4.13). While this test is more conservative than a paired test, e.g. the Wilcoxon signed-rank test, we used this model in order to take into account the standard errors in the heritability estimates when testing for the significance of the age effect on heritability. We now perform a Wilcoxon signed-rank for the difference in heritability between the two ages. As expected, the p-value for the difference in heritability with age based on the Wilcoxon signed-rank test for paired data is more significant, i.e. p= 5.56 x 10-14, than the p-value based on the Wald test, i.e. p=2.25 x 10^-3^.

We have updated Figure 3D (also shown below) to a histogram of the heritability differences (instead of ratios, to avoid problems of division by zero when heritability estimates are close to zero) and report the Wilcoxon p-value for the significance of the difference.

We also have made the following updates to the text (new text in bold below)

Page 7- Line 175-179: “We estimated the cis heritability for each gene, i.e. the proportion of expression variance explained by cis SNPs, at each age using bivariate linear mixed models. Consistent with results above, we found a small but statistically significant decrease in average cis heritability with age **(Wilcoxon signed-rank test; median(h80^2 -h70^2) = -1.2%, p-value = 5.56 x 10-14);** the average cis-heritability decreased from 18% at age 70 to 17% at age 80.”

Legend of Figure 3D: “**Difference in expression cis-heritability between ages. We see a statistically significant decrease in cis-heritability with age (Wilcoxon-signed rank test; Median(h^2_{80}-h^2_{70})= -1.4%, p-value=5.56x10^{-14})**”

9. Pi is between [0,1]. It is not the allelic ratio but "the proportion of reads supporting the reference allele". It could be called "reference allele proportion".

We thank the reviewer for this suggestion. We now use the term "reference allele proportion" throughout the manuscript .

10. The moderate correlation in Fig 4A can be explained by noise as ratios are unstable especially close to 0.5 where count-based proportions have largest variance. This should be noted since the text now might suggest this is due to an age-related change.

We thank the reviewer for pointing this out. This plot is meant to show consistency of reference allele proportions with age. We now show the correlation based on sites with higher read depth (supported by at least 100 reads) to show that reference allele proportions between the two ages are largely consistent. We have also updated Figure 4A and it’s legend. We have updated the results section as follows:

Page 8 - Lines 199-201: “We investigated transcriptome-wide patterns of allele-specific expression (ASE) with age. **At the global level, reference allele proportions within an individual were largely consistent (Spearman's ρ =0.73; Fig. 4A)**. Moreover …”

11. In discussion, p.9 line 30: argument of biological age differing from the chronological age also applies to the duration. 10 years apart for one individual does not correspond to 10 years part for another individual. So, the longitudinal design only mitigates this issue.

This is indeed the case. However, the argument of biological versus chronological age referred to the individual-specific analyses (expression outliers); where each individual can have their own aging trajectory. We have modified the discussion to clarify this (new text in bold below)

page 10- lines 242: 247: “Because the rate of aging varies among individuals, humans become increasingly different from each other with age. Thus, chronological age fails to provide an accurate indicator of the aging process. **Longitudinal studies offer a better understanding of the aging process by studying the same individuals throughout their lifespan. By collecting serial assessments, individuals can be compared to their own measurements at different time points rather than individuals of different ages from different environments.**”

12. Figure S6: Please add an explanation of the red coloring of the gene names in panel A-C.

We thank the reviewer for pointing this out. The red color corresponds to immunoglobulin-related outlier genes for the individual with the strongest expression-increasing outlier and genes related to proteasomal protein catabolic process for the individual with the strongest expression-decreasing outlier. We have now added this explanation to the Figure legend.

# Reviewer #2:

**Major Comments:**

1. One potential problem in studies that compare gene expression or other variables between two groups is the presence of batch effects that may create false positives. The manuscript overall presents careful analyses in assessing background noise and potential technical variables. However, there is no information presented on the strategy utilized to prevent any batch effect due to RNA extraction and library preparation. Was the study design balanced between age groups, with respect to these technical aspects?

We thank the reviewer for pointing out this important omission from our study design description. Indeed, study design is crucial to longitudinal studies to avoid confounding effects. Blood samples from each individual at each age were frozen immediately upon collection (in 2001-2004 for age 70 samples and 2011-2014 for age 80 samples). The RNA extraction, library preparation, and sequencing for the two samples from each individual was always performed in pairs and by the same technician. We have now added this information to the Material and Methods section (new text in bold):

page 13, lines 349-351 (new text in bold): “Gene expression was quantified for 65 individuals at both ages (130 samples). **The RNA extraction, library preparation, and sequencing for the two samples from each individual was always performed in pairs and by the same technician to minimize confounding effects.** Total RNA was extracted from …”

2. Another important covariate is sex. No information is provided about the sex of the samples and sex is not included as a covariate in the model, despite using both the X and Y chromosome in the differential expression analysis. How did the authors handle this important covariate? Ageing is a biological process that presents important differences between men and women. It would be interesting to see an analysis of sex x age effects on gene expression, if enough samples were included from each sex.

There are 30 males and 35 females in our sequenced sample. We included sex in our analyses only to account for noise in expression due to the sex effect. Specifically, we modeled the effect of sex on gene expression across the ages using a linear mixed model regression and found that sex explains 0.42% of expression variance (median across genes, Table S1 and Figure S3). Using the π1 statistic, we estimated that expression levels of ~15% of tested genes are associated with sex (Table S1). We included sex as a covariate in the differential expression analysis by age analyses that corrects for all measured factors of expression (Figure S5A, second bar in the bar plot). Moreover, sex is indirectly included as a covariate in the analyses correcting for hidden factors since it is partly correlated with these hidden factors (Figure S3A). Since the focus of the paper was on the effect of age on expression and genetic regulation of expression, we did not include any age x sex analyses. Indeed, we believe that the topic of age x sex interaction is very interesting and are working on future projects in this direction.

3. In Figure S1, it appears clear that the distribution of RIN is different between the two age groups. Indeed, RIN is one of the most important variables included in the gene expression model. Despite accounting for RIN, it is possible that residual differences in RNA degradation may account for some of the DE genes observed between the two age groups. Please include some additional analyses demonstrating that DE genes are not enriched for long genes or a 3' bias.

We thank the reviewer for this suggestion. We tested for differences in median gene length and 3’ bias between DE and non-DE genes. We did not find any large differences in length (Wilcoxon rank sum test with continuity correction; median length non-DE - median length DE = 1618; p-value = 0.02967) or 3’ bias (Wilcoxon rank sum test with continuity correction; median 3’ bias non-DE - median 3’ bias DE = -0.0029; p-value = 0.5611) between DE and non-DE genes. To keep the manuscript at a reasonable size we did not make any changes to the manuscript regarding this analysis in the revision. If the Editor and Reviewers believe that it would be useful, we are however happy to add it to the supplement.

4. A subset of DE genes is reported to be associated with complex traits. What are the complex traits the genes are associated to? Even though a full list is reported in the supplementary table, it would be helpful to include a summary of the most represented traits and interpret the biological significance of these results in the context of ageing.

We thank the reviewer for pointing this out. We have now included more information about these traits and their biological significance in the context of aging (new text in bold below).

Page 4- lines 67-84: “Moreover, 18 of these DE genes are previously known to be associated with complex traits, where gene expression levels modulate disease risk in whole blood (Sup Methods and Tab. S6). **For most of these genes, aging pushes expression toward the direction that is associated with increased disease or higher levels of risk factors. For example, the C allele in rs7941030 is associated with reduced expression of *UBASH3B*, which is down-regulated with age in our study, and increased cholesterol levels [Liu et al 2017 Nat Genet 49(12):1758-1766]. Thus, our results indicate that aging affects *UBASH3B* expression in the direction that is associated with increased total cholesterol. For a few genes, aging pushes expression toward the direction of decreased disease or risk factor, particularly for blood pressure. This negative relation between aging and blood pressure is most likely due to the fact that several of the individuals in our sample began using blood-pressure lowering medication between age 70 and 80; which in turn results in a significant decrease of both SBP (Wilcoxon signed rank test; Median SBP80-SBP70 = - 5.00, p-value = 0.04) and DBP (Wilcoxon signed rank test; Median DBP80-DBP70 = -5.99, p-value = 8.251e-05) with age. Finally, it should be noted that most of the traits associated with DE genes are cardiometabolic, e.g. triglycerides, total cholesterol and blood pressure. This is likely at least partly due to the better availability of publicly available well-powered genetic association studies of metabolic traits as compared to other age-related disease (e.g. cancer, neurological diseases, etc)**.”

In addition, we have modified Table S6 to include effect size direction for GWAS and eQTL risk allele as well as their p-values for the association.

5. Are DE genes enriched for eQTLs? Are they under stronger/weaker genetic control, compared to non-DE genes?

We do not see a significant difference in the number of eGenes between DE and non-DE genes; 6.7% of DE genes are eGenes in at least one of the two ages, as compared to 7.3% of non-DE genes (Pearson's chi-squared test for equality of proportions, p-value = 0.4745). In addition, we do not see considerable differences in the distribution of eQTL effect sizes for DE and non-DE genes (see distribution at age 70 below, age 80 results are the same); as expected, the average eQTL effect size for both DE and non-DE is close to zero (-0.0003735130 vs 0.0001796036).

We also contrasted the eGenes with the genes that showed DE by age; 7.2% of the genes with eQTLs at both ages and 9.7% of the genes with eQTLs only at age 70 showed significant differences in expression by age.

We have made the following modifications to the manuscript in order to discuss the overlap of differential expression and eQTL results:

Page 8- lines 189-192: “**We contrasted the eGenes with the genes that showed DE by age; 7.2% of the genes with eQTLs at both ages and 9.7% of the genes with eQTLs only at age 70 showed significant differences in expression by age.** *GTF2H1* is the only gene mentioned above that showed both a loss of genetic regulation and DE with age.”

6. Are the different statistical tests well calibrated? Please include QQ-plots for the p-values from the DE and differential splicing analyses and also for the eQTL and sQTL analyses.

We show below QQ-plots of the p-value distribution from the DE and DS by age analyses as well as the QTL analyses. Note that, in contrast to genome-wide association analyses of complex trait where we only expect a small number of significant hits, in transcriptome analyses, we expect a much larger number of hits, especially for the QTL analyses.

To keep the manuscript supplement at a reasonable size and since the interpretation of these QQ-plots needs some explanation (as they are not expected to look like QQ-plots from GWAS), we did not include this figure in the revision. If the Editor and Reviewers believe that it would be useful, we are however happy to add it to the supplement.

7. As mentioned above, it is surprising that the authors do not investigate if there are differences in methylation with age. Maybe such differences (if found) could explain some of the mechanisms underlying differential gene expression.

This is a really good suggestion and it is something that we also wanted to investigate. However, while the study design for the expression analyses is well balanced, i.e. the two samples of each individual were always processed together, the methylation study was not, i.e. all the age 70 samples were sequenced together and separately from the age 80 samples, making any differential analyses based on methylation challenging.

8. For the eQTL mapping analysis, were permutations run to calibrate the p-value distribution?

We did not use permutations to calibrate the eQTL p-value distribution. We performed multiple testing at the gene and eQTL level using the hierarchical testing procedure implemented in the R package treeQTL. The P-values for the Level 1 hypotheses (eGenes) are computed using Simes’ rule (Simes, 1986) on the families they index (collection of eQTL p-values for each gene). This summary of the evidence for the global null hypothesis is relatively robust to dependence (Benjamini and Heller, 2008). We have modified the Methods section of the manuscript to describe the eGene calling as follows (new text in bold):

page 16, lines 444-450: “We mapped eQTLs at each age using the linear regression models implemented in the *MatrixEQTL* R package. For the analysis correcting for measured/hidden factors, we include the measured/hidden factors as covariates. **To call eGenes at each age, we performed multiple testing at the gene and eQTL level using the hierarchical testing procedure implemented in the R package TreeQTL controlling the FDR at 1%, both at the gene and gene-SNP level. The p-values for the Level 1 hypotheses (eGenes) were computed using Simes' rule (Simes, 1986) on the families they index (collection of eQTL p-values for each gene). This summary of the evidence for the global null hypothesis is relatively robust to dependence (Benjamini and Heller, 2008).** The final number and list of eGenes at each age was obtained using the number of expression hidden factors that maximized discovery at each age, i.e. 10 hidden factors (Fig. 3A). “

9. Age-specific eQTL mapping. This is the most problematic aspect of this manuscript. A formal linear model to test for age x genotype effect is needed to convincingly demonstrate that genetic regulation of gene expression changes over time. While the method employed is valid to identify eQTLs that replicate across age groups, the 20% FDR threshold is too strict to confidently determine the absence of genetic regulation on gene expression. Lack of replication doesn't necessarily imply age-specific effects. It would also help to see a plot that compares the genetic effect (beta) between ages, similar to the plot for pi in figure 4 A. A similar issue applies to the sQTL analysis.

We thank the reviewer for pointing this out. Since we have a longitudinal design for this study, testing for the interaction effects would require a mixed model with an individual-specific random effect to model the correlation due to shared genetics and environment of each individual. Ignoring such correlations (e.g. by fitting the standard linear regression models implemented in MatrixEQTL) would lead to increase standard errors of the eQTL x Age effect sizes, compared to a mixed model, and thus reduced power to detect interaction effects. Unfortunately, mixed models are computationally intensive and are hard to scale for eQTL mapping.

As suggested by the reviewer, we compared the effect sizes at each age for the genes with eQTLs at both ages and the genes that showed loss of genetic regulation with age. The plot below shows the eQTL effect sizes for all genes with eQTLs at both ages (in black) and the effect sizes for the genes with age eQTLs only at age 70 (in dark red). The dotted line indicates the identity line (x=y). The solid grey and black lines indicate the regression line for genes with eQTLs at both ages and genes with eQTLs only at age 70.

For genes with eQTLs at both ages, the eQTL effect sizes at age 70 are significantly larger, on the absolute scale, than the eQTL effect sizes at age 80 (Wilcoxon-signed rank test; (pseudo-)median(|beta80|-|beta70|)= -0.0043, p-value < 2.2e-16, calculated across 209,677 significant gene - SNP pairs). Moreover, for genes with eQTLs only at age 70, the effect sizes at age 70 are significantly larger than the effect sizes at age 80 (Wilcoxon-signed rank test; (pseudo-)median(|beta80|-|beta70|)= -0.25, p-value < 2.2e-16; calculated across 6,015 significant gene - SNP pairs at age 70), indicating that our observation is unlikely to be only due to increased technical noise. We now include this plot as part of the main figure presenting the eQTL results (Figure 3C).

We have made the following modifications to the manuscript to discuss the comparison of genetic effects across ages (new text in bold).

Results section - page 7, lines 165-172: “**We compared the eQTL effect sizes at each age for genes that are eGenes at both ages (shared) and genes that are eGenes only at age 70. For shared eGenes, the difference in eQTL effect sizes between the two ages is small but significant (Wilcoxon-signed rank test; Median(|β80|-|β70|)= -0.0043, p-value < 2.2e-16) due to the large number of significant eQTLs from which the difference is calculated (approximately 210K). In addition, for genes that are eGenes only at age 70, the effect sizes at age 70 are significantly larger than the effect sizes at age 80 (Wilcoxon-signed rank test; Median(|β80|-|β70|)= -0.25, p-value < 2.2e-16).** To compare the effect sizes genome-wide, we estimated the *cis* heritability for each gene ... ”

Figure 3C and its legend: “**Scatter plot of eQTL effect sizes at each age for genes that are eGenes at both ages (shared) and genes that are eGenes only at age 70. For shared eGenes, the difference in eQTL effect sizes between the two ages is small (Wilcoxon-signed rank test; Median(|β80|-|β70|)= -0.0043, p-value < 2.2e-16). On the other hand, for age 70 eGenes, the effect sizes at age 70 are significantly larger than the effect sizes at age 80 (Wilcoxon-signed rank test; Median(|β80|-|β70|)= -0.25, p-value < 2.2e-16).**”

We also compared the sQTL effect sizes (see Figure below) and observed similar results, i.e. no difference in sQTL effect sizes between age 70 and age 80 for introns with sQTLs at both ages (Wilcoxon-signed rank test; Median(|β80|-|β70|)= 0.01, p-value = .999) while the sQTL effect sizes at age 70 were significantly larger than those at age 80 for the introns with sQTLs only at age 70 (Wilcoxon-signed rank test; Median(|β80|-|β70|) = -0.47, p-value < 2.2e-16).

We now include this as Figure 5D in our manuscript as well as make the following changes to the manuscript:

Results section - page 10, lines 267-273: “**We compared the sQTL effect sizes at each age for introns that are sIntrons at both ages (shared) and introns that are sIntrons only at age 70. For shared, sIntrons we do not observe a significant difference between the two ages (Wilcoxon-signed rank test; Median(|β80|-|β70|)= 0.01, p-value = 0.99). On the other hand, for introns that are sIntrons only at age 70, the effect sizes at age 70 are significantly larger than the effect sizes at age 80 (Wilcoxon-signed rank test; Median(|β80|-|β70|)= -0.25, p-value < 2.2e-16).**”

10. What is the interpretation of a difference in 1% of the heritability of gene expression across age groups?

The proportion of expression variance accounted by genetics decreases by 1% with 10 years of aging, which is consistent with the small decrease in effect sizes observed at the later age. It also suggests that, in order to get estimates of the heritability of gene expression, one should also include interaction effects with age. Absence of interaction effects has also been proposed in GWAS studies as part of the reason behind the missing heritability (cite the Or Zuk paper).

In terms of biological mechanism, increasing environmental noise or accrual of somatic mutation may underlie these reductions in heritability. We note in the paper:

page 12, lines 308-311: “The high correlation of genetic effects and the increase in allelic imbalance with age suggests that increasing environmental variance as opposed to decreased genetic variance underlies a component of the reduction in heritability and loss of genetic effects.”

11. Please include more details on the different analyses of ASE at the population and individual level using EAGLE.

We thank the reviewer for pointing this out. We have now included a more detailed description of the EAGLE2 model for the population- and individual-level different analyses of ASE (new text in bold)**:**

page 18, lines 495-507 “At the local level, we test for population- and individual-level differences in ASE with age using the beta-binomial generalized linear (mixed) models implemented in EAGLE (v2.0). **In short, for each exonic SNP, we model the alternative allele count for heterozygous individual i, at age t using a Beta-Binomial distribution, i.e. y_it ~ BB(n_it, σ(φ_i * β_t), c), where n_it is the total number of reads for i in t, σ() is the logistic function, φ_i represents the phase between the causal cis-SNP and the exonic SNP and is treated as a latent variable taking values in {-1,+1}. We learn a prior π = P(φ_i=+1) across all individuals and marginalize (sum) over the possible values of π. β_t is the effect if age on the reference allele proportion. Last, c is the concentration parameter which we learn per exonic SNP using maximum a-posteriori probability estimation with a Gamma(1.0001,1e-4) prior. For analyses at the population level (across multiple heterozygous individuals) we adapted EAGLE to handle repeated samples from the same individual, i.e. y_it ~ BB(n_it, σ(φ_i * β_t + u_i), c), where u_i ~ N(0,v) is a per individual, per exonic SNP random effect. We use variational Bayes EM to approximately integrate over all u_i while optimizing with respect to the other parameters. EAGLE2 uses the Stan probabilistic programming language [Carpenter et al 2017]**”

12. In the ASE analysis, was the entire gene haplotype considered or each individual SNP was considered independently?

For both population- and individual-level analyses, we did the analysis for each individual exonic SNP independently. We have now update the Material and Methods section describing the model used to mention that the analyses were done for each exonic SNP (see new text in the previous comment).

13. Discussion. "Here, we report a global reduction in genetic control and a reduction in gene expression heritability with age." This is a strong statement and the results provided do not convincingly support it. Less strong language should be used.

We have changed the sentence to the following: “Our results indicate a modest but significant global reduction in genetic control and gene expression heritability with age.”

**Minor Comments:**

1. Pg. 7 Line 54: This should be Fig. 5A instead of Fig. 5B.

We thank the reviewer for pointing out this typo. We have now corrected this.

2. This sentence is unclear: "Moreover, 18 of these DE genes are previously known to be complex-trait associated genes where gene expression levels modulate disease risk in whole blood (Sup Methods and Tab. S4)." Pg. 3 Line 50. Please re-write: Moreover, 18 of these DE genes are associated with complex-traits (reference) where gene expression levels in whole blood modulate disease risk (Sup Methods and Tab. S4).

We thank the reviewer for this suggestion. We have re-written the sentence as suggested on page 3:

**“Moreover, 18 of these DE genes are associated with complex traits for which gene expression levels in whole blood modulate disease risk (Sup Methods and Tab. S4)”**

3. Figure 1C, 2B-C. The triangles are not easy to distinguish. I suggest using 2 different colors but same shape for the up and down regulated genes. Or 2 different shapes that are easier to distinguish.

We thank the reviewer for this suggestion. We now use two different shapes (circles and squares) in all these figures.

4. In figure 4D, one individual goes in the opposite direction for the first 2 SNPs. What's the interpretation?

Figure 4D shows individual-specific differential ASE effects with age and reflects the fact that each individual has their own trajectory of aging (note that these two exonic SNPs are both in CD52).

5. Please check that supplementary tables and figures are numbered in the order they are referenced in the text.

We thank the reviewer for pointing this out. We have now corrected all discrepancies in the reference of supplementary tables and figures.

**Second round of review**

**Reviewer 1**

The authors addressed all my points satisfactorily. I recommend acceptance of the manuscript.

**Reviewer 2**

This is a revised manuscript by Balliu et al. The authors have addressed some of this reviewer's concerns. The main issue remaining with the current manuscript relates to the analysis of age-specific eQTLs and splicing-QTLs. Without a formal analysis of age-specific eQTLs and s-QTLs, these results should be removed because they are based on incorrect analyses. Detailed comments are below:

Major comments.

The analysis of age-specific eQTLs is still incorrect. The authors have added a plot describing the effect sizes in the two age groups. There is a clear correlation between eQTL effect size at 70 vs 80 years old. Given that the manuscript does not directly test the interaction between age and eQTL effect size, this raises concerns that some of the age-specific eQTLs are a result of lack of power at detecting eQTLs at age 80.

The argument of computational challenge in applying a mixed effect model to identify age-specific eQTLs is not acceptable as other studies have performed similar analyses with a similar paired study design in the past. Please see (Alasoo et al, 2018, Nat Genet) for an example of the procedure used. Essentially, the lead eQTL for each gene is used in subsequent interaction eQTL analysis. Using FDR thresholds to define age-specific eQTLs is incorrect and 10% or 20% is not a liberal FDR to identify age-specific eQTLs. In addition to the approach recommended above, age-specific eQTLs should also be identified by mapping the log fold change across ages. This is equivalent to a paired analysis and it is commonly used to identify context-specific eQTLs, when a paired study design is available, similar to the one used in this study.

Minor comments.

The numbers of males and females should be included in the text to give readers a more complete picture of the study sample.

The authors seem to suggest that medication use between 70 and 80 led to spurious overlaps of DE genes with blood pressure GWAS associations. Could the other overlaps (specifically with cholesterol) be due to confounding with medications?

The study design information about the methylation data is potentially useful for readers who want to use this dataset, so please consider adding it to the methods section.

**Authors Response**

**Point-by-point responses to the reviewers’ comments:**

We would like to thank the editor and reviewers for their time and comments.

# Reviewer #2

**This is a revised manuscript by Balliu et al. The authors have addressed some of this reviewer's concerns. The main issue remaining with the current manuscript relates to the analysis of age-specific eQTLs and splicing-QTLs. Without a formal analysis of age-specific eQTLs and sQTLs, these results should be removed because they are based on incorrect analyses. Detailed comments are below:**

## Major comments.

**The analysis of age-specific eQTLs is still incorrect. The authors have added a plot describing the effect sizes in the two age groups. There is a clear correlation between eQTL effect size at 70 vs 80 years old. Given that the manuscript does not directly test the interaction between age and eQTL effect size, this raises concerns that some of the age-specific eQTLs are a result of lack of power at detecting eQTLs at age 80. The argument of computational challenge in applying a mixed effect model to identify age-specific eQTLs is not acceptable as other studies have performed similar analyses with a similar paired study design in the past. Please see (Alasoo et al, 2018, Nat Genet) for an example of the procedure used. Essentially, the lead eQTL for each gene is used in subsequent interaction eQTL analysis. Using FDR thresholds to define age-specific eQTLs is incorrect and 10% or 20% is not a liberal FDR to identify age-specific eQTLs. In addition to the approach recommended above, age-specific eQTLs should also be identified by mapping the log fold change across ages. This is equivalent to a paired analysis and it is commonly used to identify context-specific eQTLs, when a paired study design is available, similar to the one used in this study.**

We would like to thank the reviewer for this suggestion. In the original version of the paper, our intention was to test for stability of genetic regulation with increasing age rather than for interaction effects specifically. This is a valid statistical approach that has been used in multiple other papers, e.g. Gaunt et al., Genome Biology (2016); GTEx Consortium, Nature (2017) etc. We did not perform any formal interaction tests since, as we mention in several places in the manuscript (lines 38-41, 176-179, 355-359), we expect that the decrease in genetic regulation with age is most likely due to an increase in environmental noise with age, rather than decrease in genetic variance. Also, as the reviewer points out this would manifest as decreased statistical power to detect e/sQTLs at age 80. We do think that this decrease in genetic regulation as a result of increase environmental noise is an interesting biological phenomenon that has been previously observed in model organisms [Viñuela et al., Genome Research 2010; Martinez-Jimenez et al., Science 2017], as well as for methylation QTLs early in human development [Gaunt et al., Genome Biology 2016]. During the review process, our intent and message became a bit unclear as a result of the addition of the test for the difference in eQTL effect sizes between the two ages. In addition, our use of the term “age-specific eQTLs” and the illustration of genes that lose regulation with age might have also added to the confusion. To make this point clear, we have now made several changes throughout the manuscript (see below), as well as added results from QTL mapping based on the log-fold change expression and splicing across ages (to show absence of large-scale interaction effects).

Abstract (new text in blue): “… We observed that individuals are more similar to their own expression profiles later in life than profiles of other individuals their own age. We identified 1,291 and 294 genes differentially expressed and alternatively spliced with age, as well as 529 genes with outlying individual trajectories. Further, while we observed a strong correlation of genetic effects on expression and splicing between the two ages, 7.8% and 9.6% of tested genes show a reduction in genetic associations with expression and splicing in older age, ~~with impacted genes enriched in DNA repair pathways~~. These findings demonstrate that, although the transcriptome and its genetic regulation is mostly stable late in life, a small subset of genes is dynamic and is characterized by reduction in genetic regulation, most likely due to increasing environmental variance with age.”

Title of section 2.3 (new text in blue): “Age-specific genetic regulation of gene expression across the transcriptome” was changed to “Stability of genetic regulation of gene expression across the transcriptome with age”

Lines 148-167 (new text in blue): “Gene expression changes in response to environmental exposures. To address the question of how stable eQTL effects are with age, we estimated the rate of replication of discovery eGenes from one age group in the other age group (Fig. 3B). To ensures low proportion of false age-specific eGenes we use a liberal FDR threshold for validation. We observed a high proportion of eGenes that replicated for each age, 92.2% (94.9%) of eGenes discovered at age 70 (80) replicated at age 80 (70) (FDR ≤ 10%), although the rate of replication was lower for eGenes discovered at age 70, compared to eGenes discovered at age 80 (Binomial proportion test; πval − πval = −2.7%, P = 3.3 × 10−3). Results remained the same for a range of discovery and replication FDR thresholds, as well as minor allele frequency thresholds (Fig. S7B).”

Lines 180-188 (new text in blue): “There are two simple explanations for the depletion of eGenes at age 80, the lower replication rate at age 70, and the small decrease in cis heritability with age. First, that the influence of genetic variation is reducing with age; or second, that the influence of environmental perturbations is increasing with age. The latter explanation is supported by the strong correlation of genetic effects as well as the lack of eQTLs for the change in gene expression with age (data not shown). Together these findings suggest that genetic effects on gene expression are largely stable late in life and that environmental perturbation is gradually increasing with age, leading to lower SNP heritability estimates and lower power to detect eQTL as age increases.”

Lines 189 – 204: ~~“We compared the eQTL effect sizes at each age for genes that are eGenes at both ages (shared) and genes that are eGenes only at age 70. For shared eGenes, the difference in eQTL effect sizes between the two ages is small but significant (Wilcoxon-signed rank test; Median(|β80| − |β70|) = −0.0043, P < 2.2 × 10−16) due to the large number of significant eQTLs from which the difference is calculated (approximately 210K). In addition, for genes that are eGenes only at age 70, the effect sizes at age 70 are significantly larger than the effect sizes at age 80 (Wilcoxon-signed rank test; Median(|β80| − |β70|) = −0.25, P < 2.2 × 10−16).~~

~~POLD3, a gene with an important role in genome stability, is among the genes with the largest loss in genetic regulation with age (Fig. 3D). Three more genes involved in the nucleotide excision DNA repair pathway, i.e. UBC, GTF2H1, and GTF2H2, also showed loss of genetic regulation with age; nucleotide excision DNA repair has been shown to mitigate the adverse biological effects of UV light in the exposed skin and is associated with age-related vascular dysfunction. In addition, GNAS, a key component of many signal transduction pathways and a known marker of age-related clonal hematopoiesis, also showed one of the largest losses of genetic regulation with age (Fig. 3E)”.~~

Title of section 2.6 (new text in blue): “Age-specific genetic regulation of alternative splicing across the transcriptome” was changed to “Stability of genetic regulation of alternative splicing across the transcriptome with age”

Lines 275 – 285 (new text in blue): “To study the stability of sQTL effects with age, we estimated the rate of replication of discovery sIntrons from one age group in the other age group (Fig. 5C). While we observed a high replication proportion at both ages, 90.4% (93.9%) of sIntrons discovered at age 70 (80) replicated at age 80 (70) (FDR ≤ 20%), the replication rate for sIntrons discovered at age 70 was significantly smaller, compared to sIntrons discovered at age 80 (Binomial proportion test; P = 2.2 × 10−2).

Last, we observed a strong correlation of sQTL effect sizes between the two ages (Fig. 5D, Spearman’s ρβ = 0.98) as well as a lack of sQTLs for the change in alternative splicing with age (data not shown). This suggest that, similar to gene expression, genetic effects on alternative splicing are largely stable late in life and that increase in environmental noise underlies the loss of sQTLs as individuals age.”

Lines 286 – 291: ~~“We compared the sQTL effect sizes at each age for introns that are sIntrons at both ages (shared) and introns that are sIntrons only at age 70 (Fig. 5D). For shared, sIntrons we do not observe a significant difference between the two ages (Wilcoxon-signed rank test; Median(|β80| − |β70|) = 0.01, P = 0.99). On the other hand, for introns that are sIntrons only at age 70, the effect sizes at age 70 are significantly larger than the effect sizes at age 80 (Wilcoxon-signed rank test; Median(|β80| − |β70|) = −0.47, P < 2.2 × 10−16).”~~

Lines 346 – 353: ~~“In addition to immune response and circadian rhythm, we observed that genes that lose regulation with age are involved in DNA repair pathways. For example, POLD3, a gene with an important role in genome stability, was among the genes with largest loss of genetic control with age. In addition, GNAS, a known marker of clonal expansion, was also among the genes with the largest loss of genetic control with age and showed a significant decrease in allele specific expression with age. Moreover, PLK3, a gene implicated in stress responses and double-strand break repair, was among the top ten genes with the strongest age-alternative splicing association.”~~

## Minor comments

**The numbers of males and females should be included in the text to give readers a more complete picture of the study sample.**

We thank the reviewer for this suggestion. We have now added sex information in the manuscript. Page 14 - Line 373 (new text in blue): “Gene expression was quantified for 65 individuals (35 females; 30 males) at both ages (130 samples).”

**The authors seem to suggest that medication use between 70 and 80 led to spurious overlaps of DE genes with blood pressure GWAS associations. Could the other overlaps (specifically with cholesterol) be due to confounding with medications?**

We thank the reviewer for this comment. Indeed, if a medication is significantly associated with aging (i.e. individuals in our sample start or stop consistently using a medication later in life), this may induce an overlap between the DE genes and complex trait GWAS genes or confound the direction of the effect of aging on expression and complex trait. We have now included this in the manuscript; page 4, lines 71-80 (new text in blue):

“For most of these genes, aging pushes expression toward the direction that is associated with increased disease risk or higher levels of risk factors … Thus, aging pushes the expression of UBASH3B towards the direction that is associated with increased total cholesterol. For a few genes, aging pushes expression toward the direction of decreased disease risk or risk factor levels, particularly for blood pressure traits. This negative relation between aging and blood pressure could be due to the fact that several of the individuals in our study began using medication that lower blood pressure between age 70 and 80; which in turn results in a significant decrease of both SBP and DBP with age (Wilcoxon signed rank test; P_SBP_ = 0.04; P_DBP_= 8.25X10^-5^ ). Other medications, including lipid-lowering, may also induce overlaps between DE genes and complex trait GWAS genes or confound the direction of the effect of aging on expression and complex traits; however, it remains a challenge to disentangle causal relationships based on these results. Finally, it should be noted that most of the traits associated with DE genes are cardiometabolic, e.g. triglycerides, total cholesterol and blood pressure.”

**The study design information about the methylation data is potentially useful for readers who want to use this dataset, so please consider adding it to the methods section.**

Thank you for this suggestion. We have now included this information in the manuscript; page 28, lines 623-629 (new text in blue):

“Signal intensities were measured with the BeadChip scanner. The 130 samples (with available RNA-Seq data) were distributed across two periods of methylation data collection, eight 96-well plates (5 plates for the 70-year old samples and 3 plates for the 80-year old samples), and forty-one 12-sample chips (24 chips for the 70-year old samples and 17 chips for the 80-year old samples).”

**Third round of review**

**Reviewer 2**

The authors have responded to all comments. As expected, the new results have weakened the conclusion of changes in genetic regulation of gene expression and splicing across age groups. While the authors are honest and rigorous in reporting the new findings, these led to a major shift in the focus of the paper. In the current version, the main finding is that genetic effects on gene regulation are overall consistent across age groups, with potential larger environmental effects later in life. Any reported difference in genetic effects across age groups seems to be circumstantial. Thus the manuscript now mainly provides a good description of genetic regulation of gene expression and splicing across age groups.

As a consequence, a revision of the title is necessary to “Genetic regulation of gene expression and splicing during a ten-year period of human aging”.

Some minor revisions are also necessary:

1. Please, align the abstract language to the new results and interpretation. Specifically please replace

“Further, while we observed a strong correlation of genetic effects on expression and splicing between the two ages, 7.8% and 9.6% of tested genes show a reduction in genetic associations with expression and splicing in older age, with impacted genes enriched in DNA repair pathways. These findings demonstrate that, although the transcriptome and its genetic regulation is mostly stable late in life, a small subset of genes is dynamic and is characterized by reduction in genetic regulation, most likely due to increasing environmental variance with age.”

With

“Further, we observed that, although the transcriptome and its genetic regulation is mostly stable late in life, a small subset of genes is dynamic and is characterized by reduction in genetic regulation, most likely due to increasing environmental variance with age.”

2. Also please report the replication rate at a nominal p-value for the replication set. This can be a panel added to figure S7B.
